# A unified-field theory of genome organization and gene regulation

**DOI:** 10.1016/j.isci.2024.111218

**Published:** 2024-10-22

**Authors:** Giuseppe Negro, Massimiliano Semeraro, Peter R. Cook, Davide Marenduzzo

**Affiliations:** 1 SUPA, School of Physics, University of Edinburgh, Peter Guthrie Tait Road, Edinburgh EH9 3FD, UK; 2 Dipartimento Interateneo di Fisica, Università degli Studi di Bari and INFN, Sezione di Bari, Via Amendola 173, I-70126 Bari, Italy; 3 Sir William Dunn School of Pathology, University of Oxford, South Parks Road, Oxford OX1 3RE, UK

**Keywords:** Natural sciences, Biological sciences, Systems biology, Data processing in systems biology

## Abstract

Our aim is to predict how often genic and non-genic promoters fire within a cell. We first review a parsimonious pan-genomic model for genome organization and gene regulation, where transcription rate is determined by proximity in 3D space of promoters to clusters containing appropriate factors and RNA polymerases. This model reconciles conflicting results indicating that regulatory mammalian networks are both simple (as over-expressing just 4 transcription factors switches cell state) and complex (as genome-wide association studies show phenotypes like cell type are determined by thousands of loci rarely encoding such factors). We then present 3D polymer simulations, and a proximity formula based on our biological model that enables prediction of transcriptional activities of all promoters in three human cell types. This simple fitting-free formula contains just one variable (distance on the genetic map to the nearest active promoter), and we suggest it can in principle be applied to any organism.

## Highlights

• We derive a formula to predict transcriptional activities of promoters genome-wide
• Predictions correlate significantly with data from different human cell lines
• This framework could be applied to predict transcription in other organisms
• 3D polymer simulations predict nuclear location of transcription factories

## Graphical abstract

**Figure:**
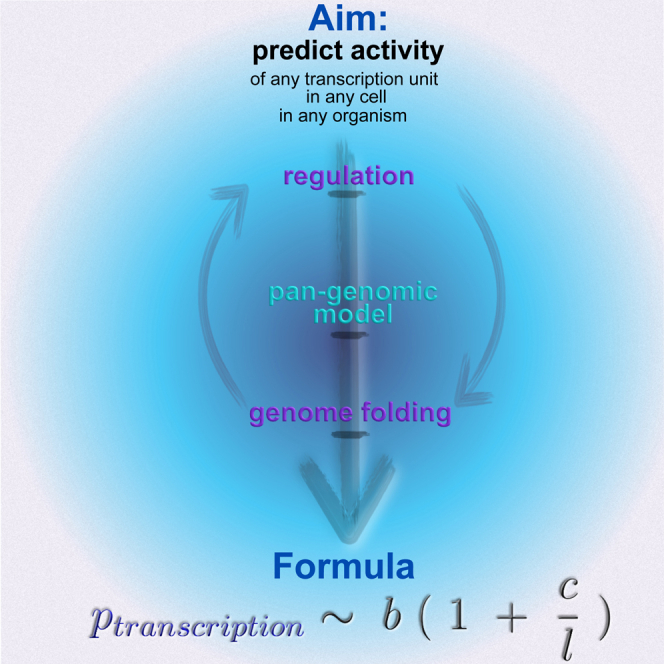

## Introduction

Our ultimate aim in this work is to predict the rate of transcription of any promoter in a given cell type. To this end, we first briefly review some key principles underlying transcriptional initiation, and a biological model based on these principles. We then present results of 3D polymer model simulations based on this model and derive a simple formula enabling prediction of the probability that a promoter might fire, which constitutes the main result of this work. We also compare results obtained using this formula with those obtained from polymer simulations and experiments.

Note that in this work we will use the term *promoter* to describe a motif governing transcriptional initiation irrespective of whether the resulting transcript is an mRNA, enhancer RNA (eRNA), or other non-protein-coding RNA; consequently, there are many more non-genic promoters than genic ones in man.^1^ When we use the term promoter, we will also assume this is active or potentially active in that cell type (as many are not active continuously and/or active in one cell type but inactive in another).

### Gene regulation: Three possible mechanisms, and two elephants in the room

A gene might be transcribed differently in two different cells in the same organism for three main reasons (Figure S1A). First, covalent structures of their DNA might differ. For example, cytosines at the 5′ ends of many human genes become methylated during development, and bases in immunoglobulin genes are shuffled during B cell maturation. However, most genes in most organisms in most cell states have similar covalent structures, so our focus on universal mechanisms means this possibility is not considered further. If covalent structures are identical, one gene might behave differently because it binds an activator or repressor, or it adopts a different 3D structure. We will argue interplay between the last two mechanisms self-organizes genomes^2^ and regulates activity.

Results of two powerful approaches dominate current thinking about gene regulation (Figure S1B). Yamanaka’s experiment points to a major role for transcription factors (TFs); over-expressing just four (Oct4, Sox2, c-Myc, and Klf4) converts mouse fibroblasts into a distinct cell type—induced pluripotent stem cells (iPSCs).^3^ Results from genome-wide association studies (GWASs) paint a very different picture. GWAS allows quantitative trait loci (QTLs) affecting any complex phenotype like the fibroblast or stem-cell state to be ranked; the sub-set influencing mRNA levels (and so transcription rates) are called expression QTLs (eQTLs) that are ordered according to the levels of poly(A)^+^ RNA determined by sequencing (RNA-seq).^4^ GWAS applied in man brought many surprises.^5^^,^^6^ First, QTLs are numerous and widely scattered, with both positive and negative effects that are individually modest. Second, QTLs rarely map to genes encoding TFs or even proteins; for example, most eQTLs are single-nucleotide polymorphisms (SNPs) in enhancers.^7^^,^^8^^,^^9^ Enhancers were originally defined as elements increasing transcription independently of orientation and position but have since been defined in many other ways.^10^^,^^11^ We will adopt a definition used by FANTOM (Functional Annotation of the Mouse/Mammalian Genome) that sees them just as an active non-genic promoters yielding eRNAs rather than mRNAs.^10^ Third, one gene is typically affected by many eQTLs/enhancers, and one eQTL/enhancer can target many genes that are often functionally related.^7^^,^^8^^,^^9^^,^^11^^,^^12^^,^^13^ Fourth, Hi-C (a high-throughput variant of 3C, chromosome conformation capture) shows eQTLs often physically contact their target genes,^13^^,^^14^^,^^15^ which points to contact (and so 3D conformation) playing a direct regulatory role.

Unfortunately, molecular mechanisms underlying eQTL and enhancer action are ill-understood, and corresponding models are complicated.^1^^,^^6^^,^^11^^,^^13^ For example, applying the *omnigenic* model^6^ to an eQTL would see an SNP altering activity of an enhancer with a consequential effect on transcription of the enhancer’s target gene (which is rarely the ultimate eQTL target). Then, once the mRNA of the enhancer’s target is translated, the resulting protein would rebalance regulatory networks in ways that depend on that target protein’s role (e.g., by influencing signaling, sub-cellular localization, ATP levels, etc.). Finally, metabolic changes would percolate back in complex ways to modify transcription of the ultimate eQTL target. Note that no attempt is made in this model to explain why an eQTL might contact its ultimate target gene.

We summarize this section as follows. Results of two powerful experiments yield unreconciled findings: Yamanaka’s result suggests regulatory networks are simple (just 4 TFs switch cell fate), but GWAS points to thousands of loci that rarely encode TFs tortuously determining phenotypes in complex post-transcriptional ways. We accept that regulation is multi-layered, but here our focus will be at the level of transcriptional initiation.

### Genome organization: DNA loops and the bridging-induced attraction

DNA loops are major building blocks of chromosomes.^2^ Those seen first in bacterial and human nucleoids, plus bacterial operons like *ara* and *lac*, were all anchored by the transcription machinery and had contour lengths of 20–200,000 bp.^16^^,^^17^^,^^18^^,^^19^ Modern techniques like Hi-C^20^ micro-C,^21^^,^^22^^,^^23^ and GAM (genome architecture mapping)^24^ confirm the presence of polymerases and TFs at anchor points. Note, however, that Hi-C misses many loops shorter than ∼200,000 bp^25^^,^^26^ and underestimates the presence of the transcription machinery at anchors.^23^^,^^27^ Most anchors in archaea and plants are also transcribed.^28^^,^^29^^,^^30^^,^^31^

**Figure 1:**
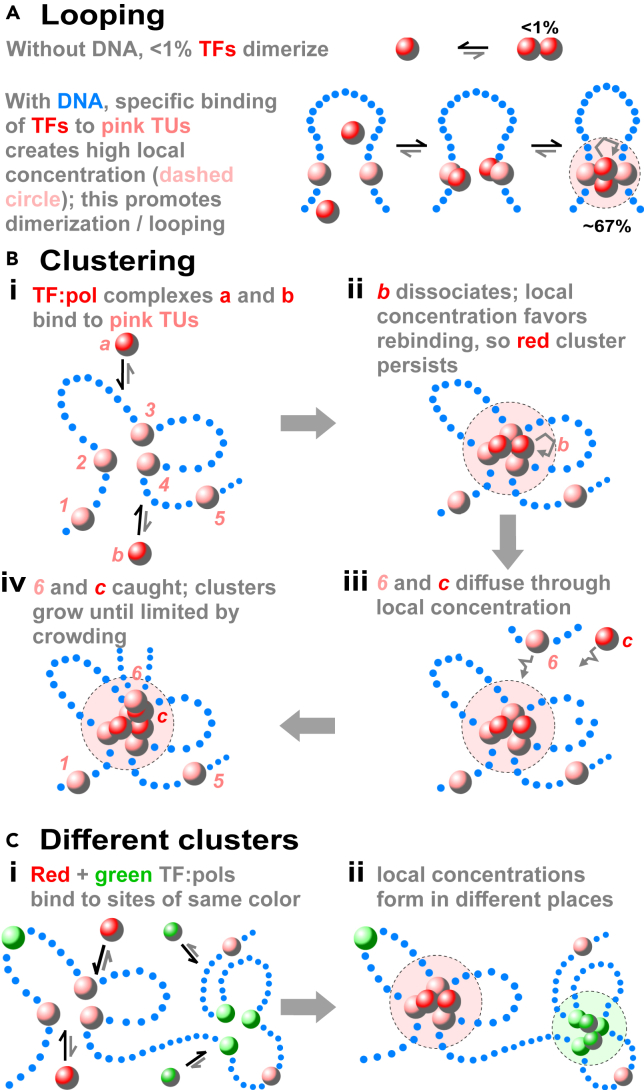
Local concentrations drive looping and clustering Strings of non-binding blue dots: DNA. Pink spheres in the string: promoters/transcription units (TUs). Red and green spheres: TFs or TF:pols able to bind reversibly to ≥2 TUs simultaneously. (A) Classical model for TF-induced looping.^32^ TF concentration = 1 nM, dimerization equilibrium constant = 10^*−*7^ M (both typical values). Without DNA, <1% TFs dimerize. When TFs bind to promoters 10 kbp apart on DNA, they often collide to give a loop. This loop tends to persist as the local concentration (dashed circle) promotes TF rebinding (gray arrow); consequently ∼67% TFs are now dimeric. (B) Clustering due to the bridging-induced attraction.^42^ TF:pols *a*, *b*, and *c* bind reversibly to TU beads *1*–*5*. No other attractive forces between TF:pols or between TU beads are specified. (i) *a* and *b* will bind. (ii) When *b* dissociates, the local concentration of binding sites enhances its chances of rebinding (gray arrow). (iii) If *6* and *c* diffuse through the local concentration of factors, both are likely to be (iv) caught. Positive feedback (capture plus little loss) now leads to ∼10 TF:pols/cluster until entropic crowding costs limit further growth. (C) Clusters of different types.^42^ (i) Red TF:pols bind reversibly to pink TU beads, and green TF:pols to light-green ones. (ii) Positive feedback drives formation of red and green clusters in different places in 3D space, as pink and light green TUs are in different places in 1D sequence space.

Loops are stabilized in four known ways. The classical mechanism involves dimerizing TFs; two TFs bind to nearby cognate sites on DNA to become trapped transiently in a local volume, and this increases the chances that they collide, dimerize, and anchor a loop^32^ (Figure 1A). Second, CTCF-cohesin complexes stabilize many mammalian loops^20^^,^^33^ (Figure S2A). While CTCF is a TF, it is not encoded by plants or bacteria, and due to our focus on universal mechanisms we only discuss it in passing. In contrast, cohesin is conserved and anchors many long loops in addition to the ones mainly discussed here,^34^^,^^35^ but it plays only a subtle role in human transcriptional regulation as knocking it down affects levels of only 23% expressed mRNAs and 1% unexpressed ones.^36^ Third, many components of the transcription machinery also possess low-complexity disordered domains^37^ (including >80% mammalian TFs plus the catalytic sub-unit of RNA polymerase II) that can coacervate into liquid droplets; then, phase separation involving components anchored at different sites can stabilize loops^38^^,^^39^ (Figure S2B). Finally, the depletion attraction is another force that arises without energy input between large particles (e.g., DNA-bound polymerases) in a solution of smaller ones (e.g., nucleoplasmic proteins) that are sterically excluded from spaces between the larger ones; its strength is in the goldilocks zone—strong enough to overcome the cost of bending DNA, yet insufficient to ensure permanent contact^40^^,^^41^ (Figure S2C). With the exception of cohesin, all known looping mechanisms involve the transcription machinery.

We now describe another mechanism that clusters many loops. Consider the polymer model illustrated in Figure 1B, where red spheres (representing TFs complexed with RNA polymerase II, and which we call TF:pols) bind reversibly to pink beads (representing promoters and associated transcription units, TUs) scattered along a string of non-binding^42^^,^^43^ (or weakly binding) blue beads (the rest of a chromosome). This model (and all subsequent ones that will be described) is based on a few assumptions and is fitting free, in contrast to most models in the field. Strikingly, Brownian dynamics simulations show that bound TF:pols spontaneously cluster to generate many associated loops through an emergent process described as a *bridging-induced attraction* (bridges create an apparent attraction). Forming such bridges inevitably increases the local concentration of binding sites, and this triggers a positive feedback mechanism that operates without energy input to recruit more TF:pol complexes. When two different kinds of TF:pol complex (red and green) are simulated, distinct red and green clusters emerge (Figure 1C). Additionally, if beads in a string representing human chromosome 19 in GM12878 cells are colored according to whether regions are active or inactive, loops, topologically-associating domains (TADs), and A/B compartments all appear without the need to invoke additional mechanisms (Figure S3).

### Interplay between genome organization and transcription: A *pan-genomic* model

Transcription can be linked to structure if one reasonably assumes that a TU is transcribed when it binds a TF:pol.^44^ Then, polymer simulations yield patterns of transcriptional activity down chromosomes that are correlated in a statistically significant way with those obtained with GRO-seq (global run-on sequencing)—arguably the gold-standard.^4^^,^^45^ Moreover, clusters resemble centers of activity seen experimentally and are called phase-separated condensates/drops/pockets,^38^^,^^46^^,^^47^ hubs,^2^^,^^48^^,^^49^^,^^50^^,^^51^ clusters,^52^ super-enhancer (SE) clusters,^53^ and transcription factories.^54^

**Figure 2:**
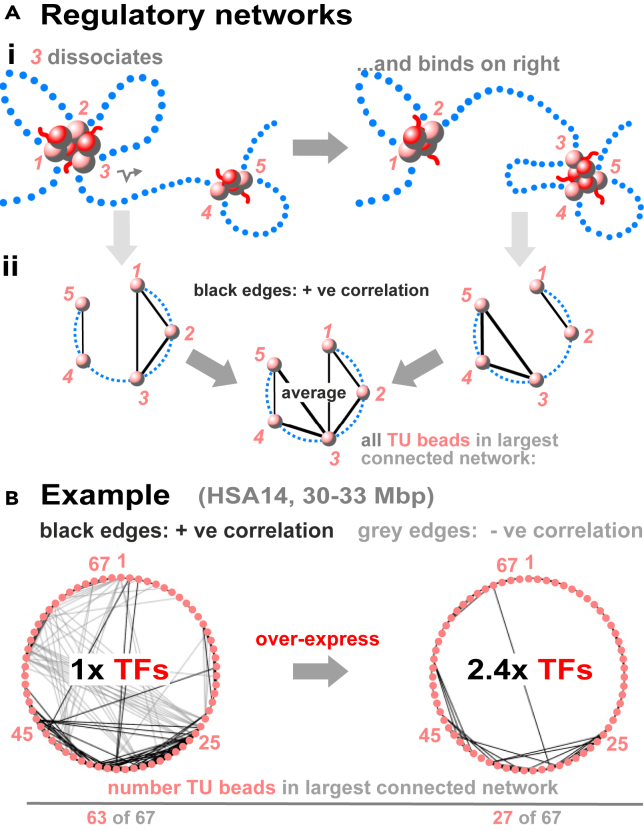
Reconciling results from Yamanaka and GWAS (A) Regulatory networks.^44^ (i) During a Brownian dynamics simulation, *3* is in a cluster with *1* and *2* and *co-transcribed* with them as all lie close to TF:pols (large red spheres). *3* now detaches from the cluster and diffuses across to initiate in the right-hand cluster so its activity now positively correlates with those of *4* and *5*. Over time, TU beads start/stop being transcribed many times and visit other clusters that appear/disappear. (ii) Contact and activity networks characterizing the two structures. Black edges indicate colocalization and positively correlated activity, with the time average in the middle. (B) Effect of TF copy number on networks seen in polymer simulations (time average given by 3 Mbp segment of HSA14 in HUVECs containing appropriately positioned TU beads, each representing 3 kbp; for data, see SI). With the *in vivo* TF concentration (*1x TFs*), many TU beads contact other TU beads and have positively correlated activities. However, TF:pols are in short supply, and binding to some TU beads necessarily decreases binding to others; this yields negatively correlated activities indicated by gray edges. The network is small-world with 63 of the 67 TU beads in the largest connected network – consistent with GWAS results. Increasing the TF concentration 2.4x simplifies the network, which we argue allows Yamanaka’s experiment to succeed.

The bridging-induced attraction naturally yields complex interaction networks.^44^ To see why, consider the structure at the top of Figure 2Ai where TU bead *3* is bound to, and transcribed by, a TF:pol. When the TF:pol terminates, *3* is free to detach and diffuse across to bind to the right-hand cluster. Later, one (or both) of these clusters may disappear, and *3* may visit other clusters when they appear. Consequently, *3* contacts many other pink TUs over time to be co-transcribed with them. A regulatory network^55^^,^^56^ can then be built (Figure 2Aii) in which each TU is represented by a node, and pairs of nodes with positively correlated activities are joined by black edges and anti-correlated ones by gray edges. Networks derived in this way from polymer simulations of human chromosome 14 in HUVECs (human umbilical vein endothelial cells) are complex (Figure 2Bi, left). Typically, black edges connect co-transcribed TUs in clusters that sequester TF:pols to reduce the likelihood that other clusters form elsewhere (giving gray edges). Strikingly, most nodes are highly connected (e.g., 63 of 67 TU beads in Figure 2Bi are in the largest connected component, left), and small-world (most nodes are inter-connected by few edges). Moreover, pink and green TU beads in Figure 1C each form their own distinct small-world networks.

To model eQTL action, TF:pol binding to each of 39 TU beads in a toy string of 1,000 beads (each representing 3 kbp) was abrogated in turn.^44^ Each knock-out rewires the whole network in a distinct way; remarkably, activities of about half the other TUs both near and far away in sequence space change slightly, as the network retains its small-world character. Moreover, introducing non-binding *heterochromatin*, binding *euchromatin*, and permanent *loops* like those anchored by cohesin rewires networks in complex and difficult-to-predict ways. All these results fit comfortably with those seen by GWAS. Significantly, they result from co-transcriptional events that would act in addition to the post-transcriptional ones envisaged by the omnigenic model.

These results lead to a *pan-genomic* model that allows reconciliation of the two conflicting views of regulation (Figure S1B). Thus far, TF:pol copy-number described in polymer simulations reflects that found *in vivo*, so TU beads do not become saturated. However, increasing copy-number dramatically simplifies networks (Figure 2B). We suggest this happens in Yamanaka’s experiment: over-expression allows the system to escape from the complex small-world networks revealed by GWAS, so just 4 TUs can play their decisive roles.^44^

We summarize thus far as follows. First, general arguments indicate that differential binding of TFs and/or folding underlie differential transcription. Second, loops are anchored by mechanisms usually involving the transcriptional machinery. Third, binding of this machinery triggers the bridging-induced attraction and clustering of TFs, polymerases, and chromatin loops. Fourth, small-world regulatory networks then emerge spontaneously from the spatiotemporal dynamics of the system. Given these basic mechanisms, evolution has a choice: life forms can either spend energy to prevent such clusters and networks forming, or they can exploit these gifts of physics. We speculate the latter happened. Possibly, the depletion attraction clustered primordial polymerases transcribing DNA. Then, when cohesins evolved they stabilized additional loops, and—once TFs appeared—the bridging-induced attraction inevitably reinforced clustering and ensured that different clusters specialized in transcribing different gene sets, with disordered motifs strengthening clustering by enhancing phase-separation. From now on we call these emergent clusters *factories*—the first name for focal sites of transcription.^54^

## Results

### A minimal model for transcriptional initiation

In car factories, local concentrations of engines and tires facilitate efficient auto production. In transcription factories, analogous concentrations underlie efficient RNA production (e.g., the concentration of human RNA polymerase II in factories is ∼1*,*000-fold higher than in the soluble pool). Consequently, the law of mass action ensures that essentially all transcription occurs in factories —as seen experimentally.^54^ Just as some car factories make Toyotas and other Teslas, the bridging-induced attraction drives formation of different transcription factories (Figure 1C) that might make, for example, inflammatory or olfactory-receptor transcripts.^49^^,^^52^^,^^57^

**Figure 3:**
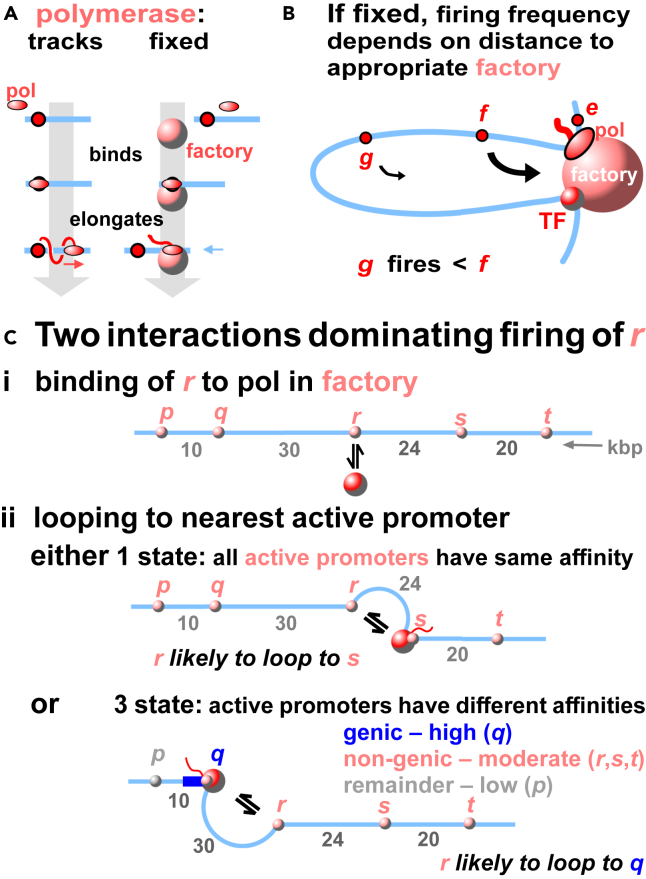
Models for transcription and gene regulation (A) Transcription cycles. The conventional model (left) has a polymerase (pol) binding to a promoter (red circle), and tracking (pink arrow) down the template; however, the resulting transcript is inevitably entwined about the helical template once for every 10 bp transcribed, and there is no known mechanism for untwining it. The alternative (right) has a polymerase binding and reeling in (blue arrow) the template as it makes an un-entwined transcript (screwing a bolt through a fixed nut provides an analogy). (B) Initiation frequency depends on distance in 3D space to a factory. Here, *f* is more likely to diffuse to the factory and fire than *g. e* also acts as an enhancer of *f* activity by tethering *f* close to the factory. (C) Feynman-like diagrams depicting two interactions dominating firing of promoter *r*. Promoters: *p*-*t*. Factory: large red sphere. (i) Binding of *r* to a factory. (ii) Forming a loop with the nearest active promoter on the genetic map. In the 1-state model, all active promoters have identical affinities for the factory. When the promoter nearest to *r* is transcribed (i.e., *s*), *r* becomes tethered close to a factory and so is likely to be transcribed. In the 3-state model, affinity of active genic promoter *q* > active non-genic ones (*r*, *s, t*) > the *other* promoter (*p*). The 2^nd^ Feinman diagram now involves the loop to the nearest genic promoter (and never to a non-genic or other one). Consequently, *r* is often tethered close to a factory transcribing *q* – and so often visits this factory to be transcribed.

In all models for transcription, initiation frequency depends on how often promoters, polymerases, and TFs interact. In ours, there are two more key features. One concerns relative movement. The traditional model sees an active polymerase tracking down its template (Figures 3A and S4). This idea stems from the perception by early biochemists of the relative size of a polymerase and its template—the smallest object (the enzyme) would move. But does it? We suggest it does not. Rather, it uses the energy released from the hydrolysis of nucleotide triphosphates to reel in its template—which has a much smaller cross-sectional area than what we now know to be a huge polymerizing complex,^38^ and so the likeliest to move end on through the viscous milieu of a cell. Evidence for this has been reviewed,^54^^,^^57^ and we here discuss one example chosen because it is often cited as the best (and to our knowledge only) evidence for the traditional model—the iconic images of Miller spreads that show polymerases caught in the act of making RNA. In these static images, each transcript appears as an extended branch in a *Christmas tree*^58^ which it is assumed is made by a moving enzyme. However, a polymerase tracking along a helical path generates a transcript entwined about the template once for every 10 bp transcribed (Figure 3A, bottom left), and not the extended and un-entwined branch seen in a spread. But if the template rotates as it is reeled in by a fixed polymerase, no entwinements result—as seen in the iconic images (Figure 3A bottom right; Figure S5, Video 1). Therefore, contrary to widespread belief, we suggest these spreads (and equivalent ones of lampbrush loops; Figure S5) provide good evidence that active polymerases do not track.

Video S1. Tracking versus fixed polymerases, related to Figure 3 The nut, bolt, and string represent a polymerase (pol), template, and transcript, respectively

The second key feature follows from the finding that most transcription occurs in factories/hubs^54^: then, initiation frequency must depend on the distance in 3D space of a promoter to a factory rich in appropriate TFs. For example, in Figure 3B, promoter *f* is tethered closer to the factory than *g*; therefore, *f* is the more likely to visit the factory and initiate. Such distance effects provide simple explanations for how regulatory motifs work. Here, *e* acts as an enhancer of *f* because it tethers *f* close to an appropriate factory. Figure S6 illustrates potential mechanisms based on this model for SEs, silencers, boundaries, insulators, eQTLs, and QTLs—as well as for other mysterious processes like transvection^59^ and the pairing of meiotic chromosomes.^60^ Note that all these motifs are transcribed when active.^1^^,^^57^ We also imagine that eQTLs and QTLs can act at the transcriptional level (and not just post-transcriptionally). Additionally, an active promoter is simultaneously any one of these motifs depending on which target gene is considered, and so it is no longer puzzling why these motifs are so similar at the molecular level.^1^^,^^54^ This model also qualitatively explains why so many contacts seen by Hi-C, micro-C, and GAM involve transcribed regions^20^^,^^21^^,^^22^^,^^24^ with contact points moving as polymerases reel in their respective templates—which is impossible to explain without additional assumptions if polymerases track (Figure S4). As expected, such contacts are sensitive to transcriptional inhibitors whereas contacts mediated solely by TFs or cohesin will be insensitive; then, it is also no longer puzzling^61^ that these inhibitors eliminate some loops but not others.

### A simple looping formula for predicting firing frequency

Deriving formulae that facilitate prediction of gene activity has a long and continuing history.^62^^,^^63^^,^^64^^,^^65^^,^^66^^,^^67^^,^^68^ For example, the deep-learning model *Enformer* shows great promise; it uses DNA sequence as input and is trained on a wide range of datasets that include DNase hypersensitive sites (DHSs), TF binding sites, and histone marks.^67^ In contrast to *Enformer*’s top-down approach, here we use a bottom-up one based on the model summarized in Figure 3B. In other words, our approach will be training-free and will not use any contact data as input.

Against the backdrop provided by GWAS that firing of any promoter is influenced by a myriad of eQTLs, we apply a strategy used by particle physicists who represent interactions dominating complex outcomes with a few Feynman diagrams. Consider potentially active promoters *p-t* on a small segment of a chromosome, each with the same affinity for a factory; this will lead to what we will call the 1-state model. We suggest two Feynman-like diagrams depict interactions dominating the probability that *r* is transcribed ($p_{trans}$): the binding of *r* to a TF:pol in a factory (Fig. 3Ci), and the stabilization of an *r*-*s* loop (*s* being the nearest promoter to *r* on the genetic map, and so likely to be the closest potential tethering point; Fig. 3Cii). Then, the two terms in this approximate formula capture interaction in these two Feynman-like diagrams (see STAR Methods for a derivation):

$$p_{trans}\left(r\right)∼b\left(1+\frac{c}{l\left(r\right)}\right)$$

Here, *b* plus *c* are two positive constants that include contributions from TF:pol concentration, promoter number, affinity of TF:pols for promoters, plus a looping probability determined using the relevant polymer model—that of a fractal globule.^69^ The only variable, l(r), is the genomic distance in base pairs from r to the nearest active promoter. Note that the two Feynman-like diagrams depicting unlooped (Fig. 3Ci) and looped structures (Fig. 3Cii, 1-state) have equal weights in the case shown where *q* and *r* are separated by 30 kbp; however, looped:unlooped diagrams have weights of 10:1, 1:1, and 1:10 for loops of 8.6, 86, and 860 kbp, respectively.

Thus far, we have assumed all promoters have identical affinities. However, GRO-seq signals at active human genes turn out to be higher than those at non-genic TUs^1^—possibly because TF:pols have a higher affinity for genic promoters. Therefore, we incorporate such a difference into the constant *b*, which then becomes a parameter dependent on promoter type $b_{r}$, and illustrate this using human chromosome 14 (HSA14) in HUVECs. We first identify all DHSs as they are such excellent markers for active promoters,^70^ but others like ATAC-seq sites could be used instead. Next, these active promoters are divided into genic and non-genic ones^71^ (using the chromatin HMM browser track). Any remaining DHSs/promoters that have not yet been included are classified as *others*. The values of $b_{r}$ for these three states (i.e., *active genic* – *b*_*g*_, *active non-genic* – *b*_*ng*_, and *other* – *b*_*o*_) are now weighted to reflect GRO-seq signals given by each type of promoter on this chromosome (data from the study by Niskanen et al.^72^). Additionally, when considering any kind of promoter, the 2^nd^ Feynman-like diagram (and term in the formula) now involves a loop to another genic promoter (and never to a non-genic or other one). Consequently, the loop between *r* and genic *q* replaces the *r*-*s* one as the 2^nd^ diagram in Fig. 3Cii, 3-state. We expect this 3-state model to perform better than the 1-state one in man, where active non-genic promoters so outnumber genic ones. Worked examples of the application of formulae to promoter’s *p*-*t* in Figure 3C are provided in STAR Methods to highlight contributions of the two diagrams. Both the 1- and 3-state formulae are *looping formulae* as they capture spatial effects by looping to the most-proximal promoter.

### Testing performance of looping formulae: Comparison with GRO-seq data

**Figure 4:**
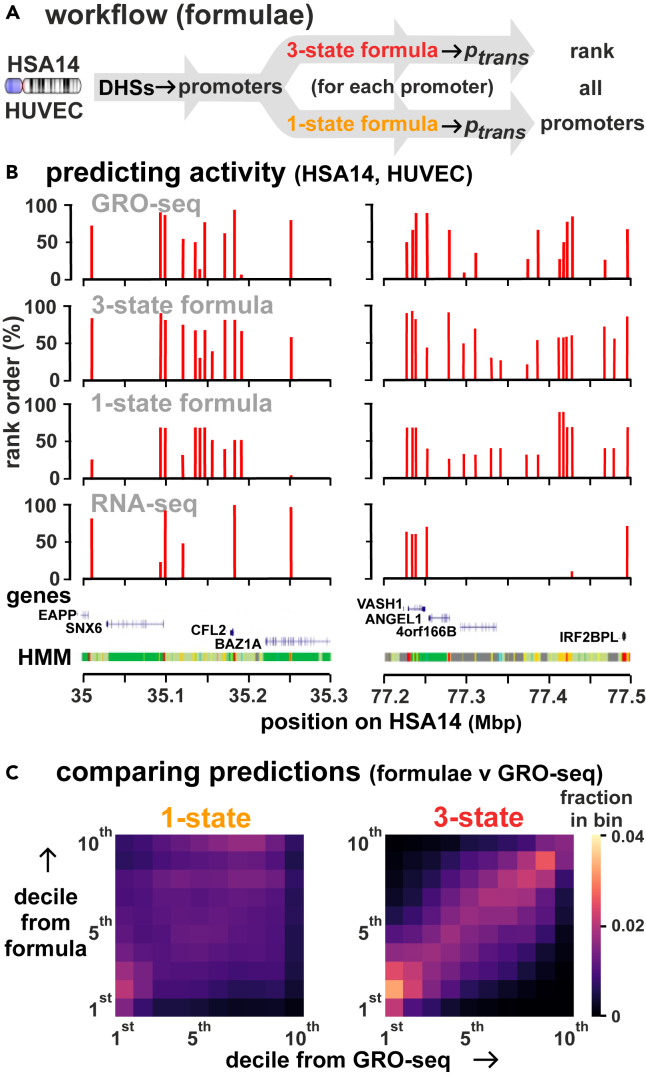
Testing the performance of looping formulae using HSA14 in HUVECs relative to results obtained with GRO-seq and RNA-seq (A) Workflow. All active promoters are identified using DHSs data (from ENCODE), their $p_{trans}$ values calculated (using *c* = 86 kbp for both formulae, and additionally *b*_*g*_, *b*_*ng*_, and *b*_*o*_ = 13.1, 3.3, and 1, respectively, for the 3-state one), and rank orders of promoters determined. 2,226 identical promoters in the 1-state case are split into 344 genic, 938 non-genic, and 944 others in the 3-state case. (B) Formulae yield activity patterns in two typical regions of the chromosome similar to those obtained by GRO-seq.^72^ Results are coarse grained into 3 kbp regions to allow comparison between datasets. The UCSC gene track excluding splice variants and non-coding genes,^73^ plus the chromatin HMM track,^71^ are included for reference (bottom). Profiles for RNA-seq miss poly(A)^-^ RNAs but include stable mRNAs; they are chosen as comparators as they are so widely used to assess transcriptional activity, and to define eQTLs. However, it is nascent RNA levels that are of prime interest here, and not steady-state ones measured by poly(A)^+^ RNA-seq. (C) Activities predicted with the 3-state formula broadly match those from GRO-seq across the activity range. Ranked promoters are binned into deciles; bin color in the heatmap reflects the fraction of all promoters found in a bin. Ten white squares on the diagonal from bottom left to top right (each with a fraction of 0.1) would represent a perfect match.

We first test the performance of each formula on HSA14 in HUVECs. We identify all promoters active on the chromosome (using DHS data), determine $p_{trans}$ for each one (for the 3-state formula using values of constant *b* determined as aforementioned), and rank $p_{trans}$ values from high to low (Figure 4A). Remarkably, patterns of activity given by the 3-state formula in two typical chromosomal segments better reflect those seen by our gold standard (GRO-seq) compared to poly(A)^+^ RNA-seq—the most widely used approach (Figure 4B). Heatmaps show that values obtained with the 3-state formula broadly match those from GRO-seq across the whole activity range from the least-to most-active (Figure 4C).

**Figure 5:**
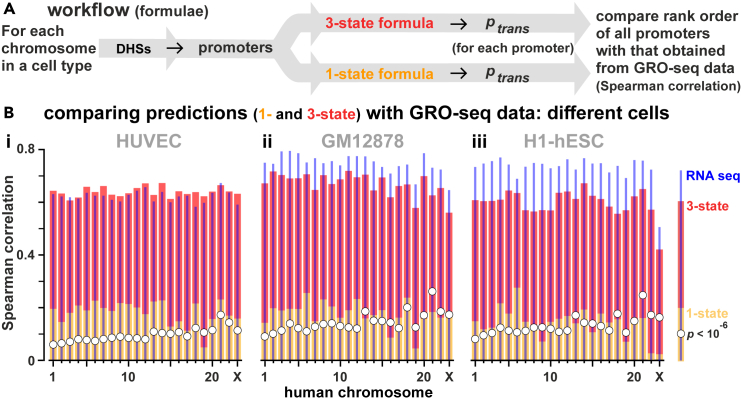
Predicting firing rates genome-wide (A) Workflow. For each chromosome, all active promoters are identified using DHSs found in that cell type, their $p_{trans}$ values calculated and ranked from high to low, and Spearman correlations between the rank order and that from GRO-seq determined. (B) Spearman correlations obtained by comparing rank orders (for each chromosome in 3 human cell types) of firing probabilities determined using the formulae (and RNA-seq) with those from GRO-seq, for each chromosome, shown as a bar plot. For the formula, we used c=86 kbp. For the 3-state formula, *b*_*g*_ = 13.1, *b*_*ng*_ = 3.3, and *b*_*o*_ = 1 (values determined using GRO-seq data for HSA14 in HUVECs are applied to all cell types). 1-state: yellow bars. 3-state: red bars. The *p* value computed measures the likeliness that correlations are obtained by chance. (i) HUVEC. The 3-state formula and RNA-seq yield roughly similar correlations. (ii, iii) GM12878 and H1-hESC. Values for *b*_*g*_, *b*_*ng*_, and *b*_*o*_ are those for HUVECs and prove to be transferable to the two other cell types; however, RNA-seq now outperforms the 3-state formula. Note that the correlations between GRO-seq and RNA-seq in the different cell lines appear to be slightly different, possibly due to technical experimental noise.

We note that the value of the Spearman correlation between ranks predicted by the 3-state formula and those found by GRO-seq is relatively insensitive to exact values of *b* and *c* (Figure S7A). For instance, for c=86 kbp, any choice of $b_{r}=b_{g},b_{ng},1$ for promoters producing mRNA, eRNA, and neither, with $5\leq b_{g}\leq 40,1\leq b_{ng}\leq 8$, leads to a Spearman correlation larger than the one between GRO-seq and RNA-seq (Figure S7Ai,ii). Additionally, for values of b used in Figure 5B, varying c between 3 and 300 kbp only changes the Spearman correlation from 0.66 to 0.62 (Figure S7Aiii; Figure S7B gives inter-chromosomal variations in values of *b*_*g*_ and *b*_*ng*_). Therefore, when applying the 3-state formula to cell types for which GRO-seq (or equivalent) data are unavailable, we expect that weightings for $b_{r}$ applied in Figure 5B can be used.

### Predicting transcription genome-wide in different cell types

We next extend this approach to all chromosomes in HUVECs, and to two other human cells for which GRO-seq data are available—lymphoblastoid GM12878, and embryonic stem cell H1. Active promoters are identified using DHSs, $p_{trans}$ values calculated, and Spearman correlations between computed rank orders compared with those determined using our gold-standard, GRO-seq (a value of 1 indicates a perfect match; Figure 5A). With all cells, our simplistic 1-state formula provides a significant correlation usually higher than that expected by chance (Figure 5Bi-iii; compare yellow bars with white circles). Remarkably, the 3-state formula slightly outperforms RNA-seq in HUVECs on all but HSA 3 and 21 (Figure 5Bi, compare red bars with blue lines). With all cells, the 3-state formula again gives high Spearman correlations around 0.6 using values for *b*_*g*_, *b*_*ng*_, and *b*_*o*_ obtained with HUVECs; clearly, these values prove to be transferable between cell types. However, now RNA-seq out-performs the 3-state formula with the other cells (Figure 5Bii,iii; compare red bars with blue lines). When comparing the 1- and 3-state formulae, the 1-state model yields higher inter-chromosomal variation (e.g., gene-rich chromosome 19 gives low correlations) than the 3-state one.

In Figure 5 we use values of *b*_*g*_ and *b*_*ng*_ obtained from GRO-seq on HSA14 and apply them to all chromosomes. As average ratios between GRO-seq signals at genic and non-genic promoters vary between chromosomes (Figure S7B), we also tested values of *b*_*g*_ and *b*_*ng*_ that are cell- and chromosome-specific. Effects are marginal, and Spearman correlations between results from GRO-seq remain around 0.6 (Figure S8). These results point to the 3-state formula providing an excellent and facile estimate of the transcriptional activity of all TUs (both genic and genic) in different cell types. Moreover, it can even be applied to cells for which no GRO-seq (or equivalent) data are available to generate weightings for *b*_*g*_ and *b*_*ng*_—as values derived from HUVECs prove to be transferable to other cell types (compare Figure 5Bi with Figure 5Bii and iii).

### Testing performance of looping formulae: Comparison with 3D polymer simulation data

**Figure 6:**
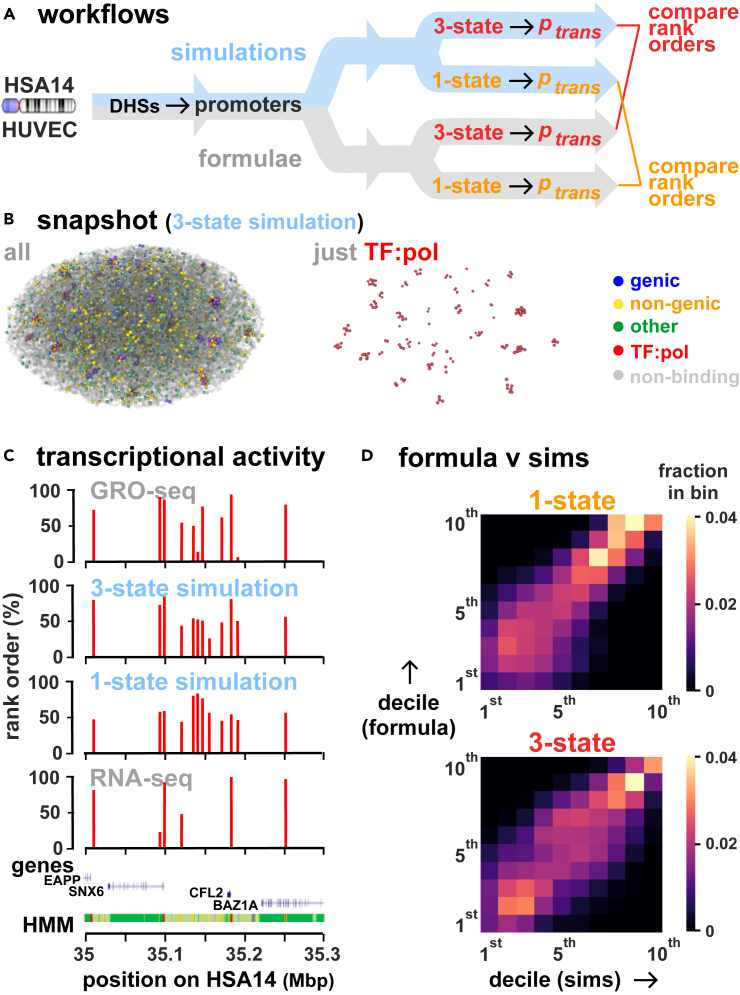
Comparing firing rates obtained using formulae and polymer simulations (A) Workflow. For HSA14 (HUVEC), all active promoters are identified using DHSs; then, using either polymer simulations or formulae, $p_{trans}$ values are calculated, ranked from high to low, and rank orders compared. (B) Snapshot from a 3-state polymer simulation (left—all beads, right—just TF:pol beads). (C) Polymer simulations yield activity patterns in a typical chromosomal segment like to those obtained by GRO-seq. Data for 1-state polymer simulations are from,^44^ they were generated in this work instead for the 3-state polymer simulations (see STAR Methods). Coarse-graining is as in Figure 4B, from where GRO-seq, poly(A)^+^ RNA-seq, gene, and HMM tracks are reproduced for comparison. Spearman correlations and *p* values with GRO-seq data are, respectively: (i) 0.32 and <10^−6^ (polymer simulations, 1 state); (ii) 0.60 and <10^−6^ (polymer simulations, 3 states); (iii) 0.62 and <10^−6^ (poly(A)^+^ RNA-seq). Another comparison can be done by binning rank data into deciles. Then, we count how many data fall in the same bin from GRO-seq and the other tracks, normalizing this count by the number expected by chance: this ratio measures how good agreement with GRO-seq is. Ratios are: (i) 1.31 (1-state polymer simulations); (ii) 1.82 (3-state polymer simulations); (iii) 1.71 (poly(A)^+^ RNA-seq). (D) Activities predicted with the 3-state formula broadly match those from polymer simulations (sims) across the activity range. As in Figure 4C, ranked promoters are binned into deciles, bin color in the heatmap reflects the fraction of all promoters found in a bin, and 10 white squares on the diagonal from bottom-left to top-right (each with 0.1 counts) would represent a perfect match.

We have seen that firing rates can be obtained from both 3D polymer simulations and formulae, and then validated by comparison with GRO-seq data. To provide additional validation (as well as a sanity check) we now complete the loop by comparing firing rates determined using formulae with those obtained from sets of polymer simulations that mirror the 1- and 3-state conditions (Figure 6A). In these Brownian-dynamics simulations, HSA14 in HUVECs is represented as a bead-and-spring polymer (a string of 35,784 beads, each of 30 nm diameter corresponding to 3 kbp) that is confined within an ellipsoid of appropriate volume^44^ (as individual chromosome territories are often ellipsoidal). Beads containing DHSs are identified as ones containing promoters, and those representing open chromatin by the presence of H3K27Ac histone-modifications. Polymer simulations performed previously^44^^,^^74^ provide 1-state data; in these, TF:pols were attracted to promoter-containing beads via a Lennard-Jones potential of 7.1 $k_{B}T$ (interaction range equal to 1.8 times bead diameter), and to beads marking acetylated regions more weakly^44^ (potential of 2.7 $k_{B}T$) – with all other beads being non-binding. For the new 3-state polymer simulations, promoter-containing beads are sub-divided into three types with different attractive potentials (i.e., genic – 7.1 $k_{B}T$, non-genic – 4.4 $k_{B}T$, and others – 3.5 $k_{B}T$), with the weakly- and non-binding beads being as for 1-state polymer simulations (see STAR Methods for more details).

Figure 6B illustrates a snapshot of a Brownian-dynamics simulation volume at steady state in a 3-state run. As expected, clusters containing TF:pols bound mainly to genic and non-genic promoters spontaneously emerge. While these clusters occur throughout the volume, most (i.e., 88.5 ± 0.5%, where the error is standard error of the mean) are found in the outer 50% of the ellipsoidal volume (Figure 6). This striking difference is in accord with results of *intron seqFISH* showing that most nascent human transcripts are found close to the surface of chromosome territories.^75^ Such a peripheral location is also consistent with the greater numbers of *trans* versus *cis* chromosomal contacts seen by ChIA-PET after pulling down pol II,^76^ and by GAM.^24^ The mechanism driving clusters to the periphery is likely to be entropic: dispersing clusters with their high DNA densities as far away from each other as possible should reduce the free energy of the system. As before,^44^^,^^74^ we determine $p_{trans}$ by measuring the amount of time a TF:pol spends bound to a bead. Both 1- and 3-state polymer simulations lead to activity patterns down chromosomes that match those seen by GRO-seq (Figure 6C). Both also yield Spearman correlations that correlate well with those given by formulae across the activity range (Figure 6D). This provides further validation of the effectiveness of the looping formulae, and that formulae can replace 3D polymer simulations when computational resources are limited, or if calculation time needs to be minimized.

## Discussion

Our aim was to predict how transcriptionally active a gene might be in any given cell under any given condition—against the background that gene regulation appears to be both simple (only 4 Yamanaka TFs switch cell fate) and complex (a myriad of eQTLs determine complex phenotypes, with few of these eQTLs encoding TFs; Figure S1B). Therefore, we reviewed a parsimonious pan-genomic model for both the organization and regulation that accommodates these very different results (Figure 3B). In this model, transcription rate depends mainly on the frequency with which a promoter visits a factory rich in appropriate TFs, and this enables us to propose simple mechanisms for how mysterious motifs like enhancers, silencers, and eQTLs all work (Figure 3B and S6). Note that these factories are unlike car factories with stable architectures; instead, they are ephemeral, morphing as TF:pols bind/dissociate, and loops appear/disappear. Moreover, chromosome conformation is unlikely ever to be the same in two daughter cells in any tissue in our bodies simply because there are so many promoters able to visit so many appropriate factories, with any active promoter influencing the activity of all others to some degree.^44^ Then, the logic developed during evolution that is embedded in DNA sequence, TF concentration, and binding frequency acts through the bridging-induced attraction to organize small-world networks of clusters and loops that determine initiation rates.

Motivated by this pan-genomic model, and by focusing on just two configurations that we suggest dominate outcomes (i.e., binding to a factory, and forming the most-likely loop), we went on to develop two variants of a simple looping formula that enable prediction of the probability that any promoter (whether genic or non-genic) is transcribed. The 1-state formula treats all promoters identically and requires as input only the number of bp to the next nearest promoter on the chromosome. The 3-state formula divides promoters into three groups that fire at different rates (i.e., genic, non-genic, plus ones not included in either of these two lists). When this 3-state formula is applied to 3 human cells (HUVECs, lymphoblasts, stem cell), it performs as well as RNA-seq and 3D polymer simulations in estimating the firing rates of all promoters across the whole activity range, and gives Spearman correlations of ∼0.6 when rank orders of firing probabilities are compared with those obtained from GRO-seq (Figures 4, 5, and 6). Consequently, these looping formulae have various general advantages. They are based on the physical forces we suggest drive genome conformation and promoter firing, they depend mainly on only one variable (distance in base-pairs to the nearest active promoter) and not on data-fitting and/or machine-learning used by other promising (but top-down) approaches,^67^^,^^77^ they are easily extended (e.g., by adding more diagrams that include *trans* contacts and/or different TF:pol complexes binding with different affinities, and by varying weightings between different diagrams), and they are general in that they can be applied in any organism (however, they can only be validated when appropriate GRO-seq or equivalent data are available). At the same time, we stress that there are some assumptions in our derivation of the looping formulae, which ultimately limit their applicability (see Limitations of the study further).

We hope our unified conceptual views of genome organization and gene regulation can be combined with others to eventually enable us to predict a gene’s activity and alter it in any desired way. The result of Yamanaka’s experiment coupled with the success of the *AlphaFold2* algorithm in solving the protein-folding problem^78^^,^^79^—which is analogous to the genome-folding problem outlined here—encourages us in our hopes. In Figure S9 we describe a roadmap that addresses a grand challenge—how one might switch the fate of any human cell in a desired way.

### Limitations of the study

In this study, we have derived a formula to predict transcriptional activity of promoters genome-wide. While the correlations of the corresponding predictions with experimental data are statistically significant, there are some limitations deriving from the approximations which we have employed. The main ones are the following. (i) We include only two diagrams, and do not include *trans* contacts. (ii) We do not include different types of TUs (for instance, binding to different types of transcription factors). (iii) In the 3-state formula, we only loop in the 2^nd^ diagram to the nearest genic promoter (however, far away it might be, and never to a non-genic or *other* one that might be closer). We anticipate that in the future it will be important to tune these assumptions to accommodate new data, and to add complexity so that a higher Spearman correlation with GRO-seq data can eventually be achieved. Additionally, it will be important to perform 3D simulations of chromosome structure and transcription genome-wide in their nuclear context, to add another dataset which can be compared to the predictions of our looping formulae.

## Resource availability

### Lead contact

Any additional information required to reanalyze the data reported in this study is available upon reasonable request from the lead contact, Davide Marenduzzo (dmarendu@ph.ed.ac.uk).

### Materials availability

This study generated no new unique reagents.

### Data and code availability

• Processed DHS peaks for HUVEC, GM12878, H1-hESC and GRO-seq data are available on-line at https://git.ecdf.ed.ac.uk/dmarendu/unified-field-theory-of-genome-organisation-and-gene-regulation.
• The LAMMPS software we used for 3D polymer simulations is freely available on-line at https://www.lammps.org. Sample additional simulation and an analysis scripts are instead available at https://git.ecdf.ed.ac.uk/dmarendu/unified-field-theory-of-genome-organisation-and-gene-regulation.

## Acknowledgments

This work was supported by the 10.13039/501100000781European Research Council (CoG 648050, THREEDCELLPHYSICS; DM). M.S. acknowledges PRIN/2020 PFCXPE from 10.13039/501100021856MUR for financial support. We thank C. A. Brackley and M. Chiang for helpful discussions.

## Author contributions

G.N., M.S. and D.M. performed the polymer simulations, and the analytical and numerical calculations with the formulae; G.N., M.S., P.R.C. and D.M. wrote the paper.

## Declaration of interests

The authors declare no competing interests.

## STAR★Methods

### Key resources table

**Table:** 

REAGENT or RESOURCE	SOURCE	IDENTIFIER
**Deposited Data**		

Processed DHS peaks for HUVEC, GM12878, H1-hESC.	Data used are given in a repository in this work	https://git.ecdf.ed.ac.uk/dmarendu/unified-field-theory-of-genome-organisation-and-gene-regulation
GRO-seq data.	Data used are given in a repository in this work	https://git.ecdf.ed.ac.uk/dmarendu/unified-field-theory-of-genome-organisation-and-gene-regulation

**Software and Algorithms**		

LAMMPS: new 3D polymer simulations of chromosome organization and transcription in HSA14 (HUVEC cells) were performed with the freely available LAMMPS molecular dynamics software.	From^80^	https://www.lammps.org
Additional simulation and analysis scripts.	This work	https://git.ecdf.ed.ac.uk/dmarendu/unified-field-theory-of-genome-organisation-and-gene-regulation

### Experimental model and study participant details

No experimental model was implemented in his study, neither any participat of any kind was involved.

### Method details

#### A looping formula relating initiation rate to distance from an appropriate factory

Here we describe how to derive the formula which we used to predict transcriptional activity genome-wide starting from the position of DNase hypersensitive sites (a TU in our formalism).

Consider a genomic segment with *N* TU beads that bind *n* TF:pol complexes; our formula will yield the probability that a given promoter in a TU, *i*, is transcribed – which we estimate as the probability that a TF:pol is bound to *i*. Many configurations contribute to this probability, but two do so significantly as they are the first steps in the firing pathway (depicted in Figure 3Cii as Feynman-like diagrams): one where *i* binds to a polymerase in a factory, and the second where *i* is tethered by the nearest active promoter to that factory. As we consider only promoters that are active or potentially active in the cell type under consideration, the Boltzmann weight of the unlooped configuration is ${n\;e}^{\beta \epsilon }\left(1-p\left(l\left(i\right)\right)\right)$, where $\beta =1/\left(k_{B}T\right)$ with $k_{B}$ the Boltzmann constant and T the temperature, ϵ is the affinity between TF:pol and *i* (which is initially assumed to be same for all promoters for simplicity in the one-state model; Figure 3Cii), l(i) is the distance on the genetic map in base pairs between *i* and the nearest promoter, and p(l) is the probability that the segment is looped (anchored by *i* to its nearest promoter, note we drop in the derivation the dependence of l on i for simplicity of notation). The Boltzmann weight of the looped configuration is ${n\;e}^{2\beta \epsilon }p\left(l\right)$, because now there are two contacts between the TF:pol and a TU.

For a confined chromosome, the relevant polymer model is that of the fractal globule, for which the looping probability can be approximated as $p\left(l\right)∼\frac{a}{l}$, with a a suitable constant.^69^ The sums of the Boltzmann weights of looped and unlooped configurations (Figure 3Cii) is then $n\;e^{\beta \epsilon }\left[1+\frac{\left(e^{\beta \epsilon }-1\right)a}{l}\right]$, and the transcriptional probability is this sum divided by the sum of weights of all possible configurations (which we denote by Z, and whose explicit form we do not require here because we will only consider rankings of transcriptional activities, so that its value drops out of the analysis). Then, the probability that *i* is transcribed ($p_{trans}$) has the form:

(Equation S1) $$p_{trans}\left(i\right)∼b\left(1+\frac{c}{l\left(i\right)}\right)$$

where b plus c are two positive constants (b includes contributions from TF:pol concentration, promoter number, and the affinity of TF-pols for promoters; c includes contributions from the affinity of TF-pols for promoters, and looping). For example, the unlooped configuration in Figure 3Cii (for which i=r) gives a weight of b, and the looped one a weight of $\frac{bc}{l\left(r\right)}$ , where l(r)=24 (i.e., distance in kbp between *r* and *s*). Note that as ranks of transcriptional activities are compared in subsequent statistical analyses, the two constants b and c become irrelevant in this case (because they divide out and do not contribute to the relative rank).

This simple formula, which we refer to as the *looping formula* to highlight the fact that it captures the effect of 3D looping to proximal promoters, can be generalized to situations where different promoters bind TF:pol complexes with different affinities, and the approach that follows is motivated by the observation that active genes in GM12878 cells were known to yield 2- to 3-fold higher GRO-seq signals than active enhancers.^1^^,^^81^ For instance, consider the case where a promoter (corresponding in our example to a DHS peak) produces either a mRNA, or an eRNA (or other non-genic RNA), or one that is in neither of these two lists. These three states are identified using the ChromHMM browser track^71^ in which active genic promoters are marked by HMM state 1, active enhancers and other non-genic RNAs by HMM states 4 + 5, and some DHSs have none of these HMM states (these will constitute our *other* class). In the *3-state model*, instead of a constant b we use $b_{i}$, a parameter dependent on 3 promoter types that apply to TUs that are *active genic* – $b_{i}=$ *b*_*g*_, *active non-genic* – $b_{i}=$ *b*_*ng*_, and *other* – $b_{i}=$ *b*_*o*_. Then, when considering any promoter *i* (whether it is genic, non-genic, or other), the loop in the 2^nd^ Feynman diagram connects *i* to the nearest genic promoter (e.g., *r* connects to genic *q* in Figure 3Cii, 3-state), and $b_{i}$ now captures *i*’s state (i.e., it is higher for such a promoter producing a mRNA, intermediate for one producing an eRNA, and smaller for the *other* class). To reiterate, when considering any kind of promoter, we only loop in the 2^nd^ diagram to another genic promoter (and never to a non-genic or other one).

We now provide worked examples where each formula is applied in turn to promoters *p-t* in Figure 3C. First, consider the 1-state formula. Constants *b* and *c* contain components capturing TF:pol and promoter concentrations, plus the affinity of TF-pols for promoters. As our aim is to compare relative values of $p_{trans}$ and determine a rank order, and as each of these components is common to every promoter, we note that both *b* and *c* are actually irrelevant in the 1-state model (they will divide out). However, in what follows we include these constants in the calculation for clarity. As the average loop length is not known for the cells analyzed, we use the average length found in HeLa^54^ (i.e., 86 kbp) and set this equal to *c*. Then, for promoter *r*, $p_{trans}\left(r\right)∼b\left(1+\frac{86}{24}\right)≃4.58\;b$ (as c=86 kbp and l=24 kbp) and values for p−t are 9.6b, 9.6 b, 4.58 b, 5.3 b, 5.3 b respectively; this gives a rank order (from high to low) of p=q,s=t,r. Now consider the 3-state formula. For promoter *r*, we note that the distance to the closest genic promoter is now l=24kbp, so that ${p_{trans}\left(r\right)\;∼\;b}_{ng}\left(1+\frac{86}{30}\right)≃3.87\;b_{ng}$. Values for p−t are $9.6\;b_{o}$, $b_{g}$, 3.87 $b_{ng}$, 2.59 $b_{ng}$, 2.16 $b_{ng}$, respectively (note that the estimate of firing of q includes only the first diagram as calculating the second requires knowing the location of the closest genic promoter which is not shown in the sketch). In Figure 4B, we use values for *b*_*g*_, *b*_*ng*_, and *b*_*o*_ = 13.1, 3.3, and 1, respectively, which gives a rank order (from high to low) of q,r,p,s,t. These values of *b*_*g*_, *b*_*ng*_, and *b*_*o*_ are based on observed average ratios between GRO-seq signals for mRNA, eRNA, and other DHS peaks in human chromosome 14.

Our theory can be developed to include additional topologies (e.g., *trans* contacts, as well as double loops as in Figure 1Bii), and different types of TF:pol (e.g., to include red plus green TFs binding to pink and light-green promoters respectively, as in Figure 1Cii). The latter is especially relevant when modeling different cell types (as in Figure S9). In this case, looped Feynman-like diagrams would connect promoters of the same type/color. Note that simulations of TFs with 5 different colors each binding with a different affinity to 5 different kinds of TU bead leads to clusters that mainly contain TU beads of the same color.^42^ Consequently, this formula can be extended to tens of different TFs when analyzing complex mammalian cell states and addressing the grand challenge outlined in Figure S9.

Note that our formula can be applied to any organism for which chromosomal positions of all promoters active in the cell type under study are available (which can be obtained, for example, using GRO-seq, ATAC-seq, or inspection of appropriate histone marks). However, a critical test of how accurately our formula (or any other approach) enables prediction of activity requires lists of relative activities of all transcription units in a genome (i.e., both genic and non-genic). While lists of relative numbers of steady-state RNAs are widely available, those of relative numbers of nascent transcripts are not (they are obtained, for example, by GRO-seq). It is for this reason that our tests use human data, but testing can be extended to other species as appropriate data becomes available.

#### Polymer physics modeling

For 3D polymer simulations (Figure 6), we used a *3-state* variant of the DHS model first discussed in^44^; note we used data from that paper for the *1-state* variant. In both variants, chromosomes are coarse-grained into bead-and-springs polymers, with 1 bead corresponding to 3 kbp (diameter σ= 30 nm), so that HSA14 of HUVEC is coarse-grained into 35,784 beads. The chromosome is confined within an ellipsoid of appropriate volume^44^ (semiaxes 22.24σ:34.24σ:41.80σ). Beads containing DHSs are identified as TUs in both variants, and those representing open chromatin by the presence of H3K27Ac histone-modifications.

Polymer simulations performed previously^44^ provide 1-state data; in these, TF:pols were attracted to TU beads via a Lennard-Jones potential given by

(Equation S2) $$U^{LJ}\left(d\right)=\left\{ \begin{array}{c} \frac{4\epsilon }{N}\left[{\left(\frac{\sigma }{d}\right)}^{12}-{\left(\frac{\sigma }{d}\right)}^{6}-{\left(\frac{\sigma }{r_{c}}\right)}^{12}+{\left(\frac{\sigma }{r_{c}}\right)}^{6}\right]\;\text{if}\;d<r_{c}\\ 0\;\text{otherwise}, \end{array}\right.$$

where d is the distance between TU and TF:pol, $r_{c}=1.8\sigma$ is the interaction range (or cut-off), $\epsilon =7.1\;k_{B}T$ is the interaction strength, and N is a normalization factor ensuring that the minimum of the potential is given by ϵ. TUs interact again according to Equation S2, but weaker, with chromatin beads corresponding to acetylated regions^44^ (ϵ= 2.7). All other chromatin beads are non-binding, so there is only excluded volume interactions between them and TF:pols^44^ (modeled via a Weeks-Chandler-Anderson potential).

For a set of 64 new 3-state polymer simulations, TU beads are sub-divided into three types with different attractive potentials: as done for the 1D model, these three states are identified using the ChromHMM browser track.^71^ TU beads corresponding to active genic promoters are marked by HMM state 1 and interact with TF:pols via Equation S2, with ϵ= 7.1 $k_{B}T\text{.}$ TUs corresponding to active enhancers and other non-genic RNAs are marked by HMM states 4 + 5, and interact with TF:pols via Equation S2, with ϵ= 4.4 $k_{B}T$. Finally, all other TUs interact with TF:pols via Equation S2, with 3.5 $k_{B}T$. Interactions between TF:pols and the weakly- or non-binding beads are treated exactly as for 1-state polymer simulations.

As is standard in these types of polymer simulations, the potential field also includes finitely extensible nonlinear elastic springs between neighboring pairs of chromatin beads and bending energy between triplets of neighboring chromatin beads. TF:pols interact repulsively via Weeks-Chandler-Anderson potentials, and so do any two pairs of chromatin beads. TF:pols switch between an active (chromatin binding) and an inactive (non-binding) state at a constant rate. The parameter values used in this work, as reported here, are the same as those used in the 1-state model studied in.^44^^,^^74^

### Quantification and statistical analysis

Data for the *formula* tracks in Figure 4B were derived as described in A looping formula relating initiation rate to distance from an appropriate factory. Comparison between outputs from our formula and GRO-seq data were performed by evaluating the Spearman’s rank correlation coefficient between these sets of values. Note that the value of the Spearman correlation between ranks predicted by the 3-state formula and those found by GRO-seq is relatively insensitive to exact values of *b*_*g*_, *b*_*ng*_, and *b*_*o*_ and *c*. For instance, for values of *b*_*g*_, *b*_*ng*_, and *b*_*o*_ used in Figure 4B varying between 3 and 300 kbp changes the Spearman correlation from 0.66 to 0.62 (Figure S7). Additionally, for c = 86 kbp, any choice of *b_g_, b*_*ng*_, and *b_o_=1*, for promoters producing mRNA, eRNA and neither, with *5≤b*_*g≤40*_, *1≤b*_*ng*_*≤8*, leads to a Spearman correlation larger than the one between GRO-seq and RNA-seq. Therefore, when applying the 3-state formula to cell types for which GRO-seq data (or equivalent ATAC-seq data) is not available, the exact weightings for *b* are unlikely to be crucial, and could be estimated based on our results or using data from close relatives of cell types for which data are available.

In Figure 5 and S8 the *p*-values with threshold $10^{-6}$ measures the likeliness that correlations are obtained by chance.

Data for the *simulations* tracks in Figure S3B were taken from^44^ for 3D polymer simulations in the 1-state (DHS) model. For the 3-state case, see Polymer Physics Modeling.
