# Supplementary material for: A unified-field theory of genome organization and gene regulation
Source: iScience. 2024 Oct 22;27(12):111218. doi: 10.1016/j.isci.2024.111218 (PMC11607604; doi:10.1016/j.isci.2024.111218)
Supplement: Document S1. Figures S1–S9 [file mmc1.pdf]

iScience, Volume *27*

Supplemental information

A unified-field theory

# of genome organization and gene regulation

Giuseppe Negro, Massimiliano Semeraro, Peter R. Cook, and Davide Marenduzzo

SUPPLEMENTARY FIGURES

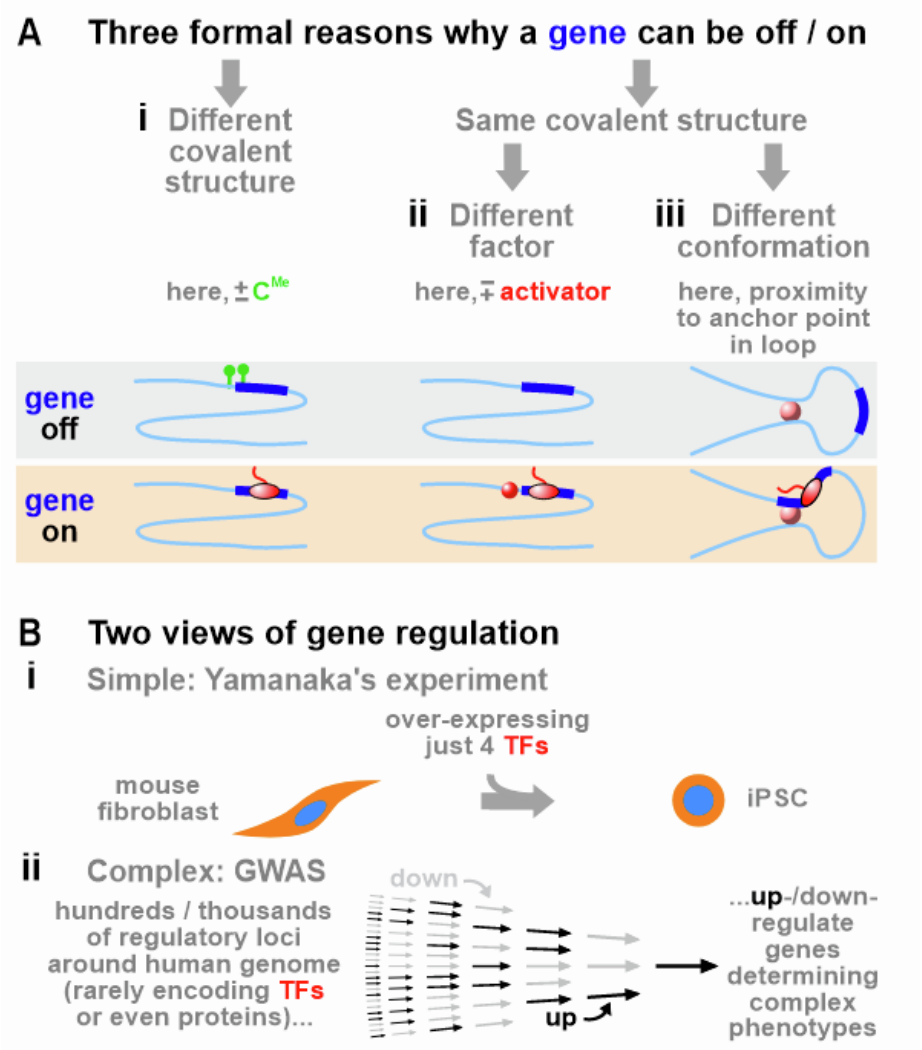

Figure S1. Ways of switching a gene on/off, and conflicting experimental results, related to Figure 1.
A. Three possibilities. Oval + wavy red line: polymerase + transcript.
(**i**) Covalent DNA structures differ in the off/on states (here, due to methylation of C residues – green lollipops).
(**ii**,**iii**) If covalent structures are identical, one gene may associate with a different factor (here, an activator – red sphere), or adopt a different conformation (here, the polymerase only initiates at the point anchoring a loop).
B. Two experimental results give conflicting views of how activity is regulated.
(**i**) Simple (from Yamanaka's experiment).
(**ii**) Complex (from GWAS).

Click here to enter text.

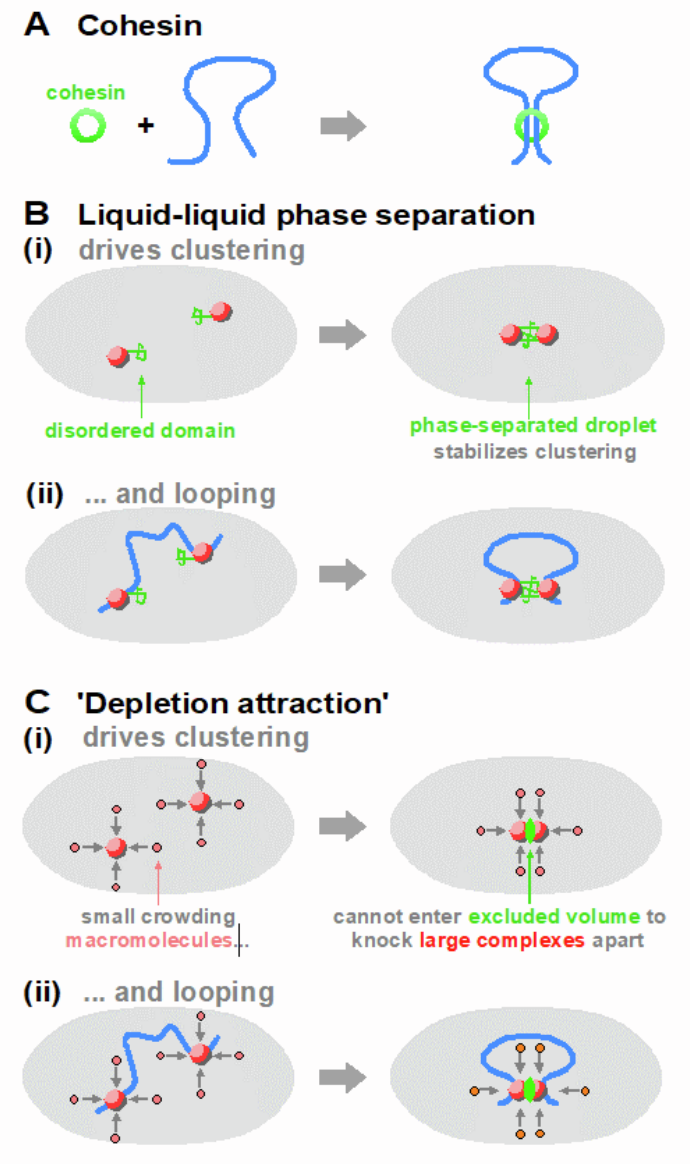

Figure S2. Major mechanisms stabilizing loops, related to Figure 1.
A. Cohesin is a ring-shaped molecule that clips on to DNA like a carabiner on a climber's rope to stabilize loops[1,2]. [The bridging-induced attraction drives cohesin aggregation[3], which is seen in mouse ES cells – with transcription opposing this[4].]
B. Phase separation.
(i) The catalytic sub-unit of mammalian polymerase II[5-8], plus initiation (e.g., MED1 with OCT4, ER, MYC, NANOG, SOX2, GATA2) and elongation factors[9] (e.g., CYCT1 of P-TEFb, DYRK1A) contain low-complexity disordered domains that can form liquid drops.
(ii) Such drops can stabilize clusters/loops[5-7,9-11].
C. The depletion attraction[12,13].
(i) In crowded cells, many small pink molecules (diameter < 5 nm) bombard (grey arrows) larger red complexes (5 − 25 nm) from all sides. If two large complexes collide, smaller molecules are sterically excluded from the green volume between the two and cannot knock them apart; consequently, small molecules exert a force on opposite sides of the two to keep them together.
(ii) When polymerases (5-25 nm) bind to DNA this force can drive clustering/looping.

Click here to enter text.

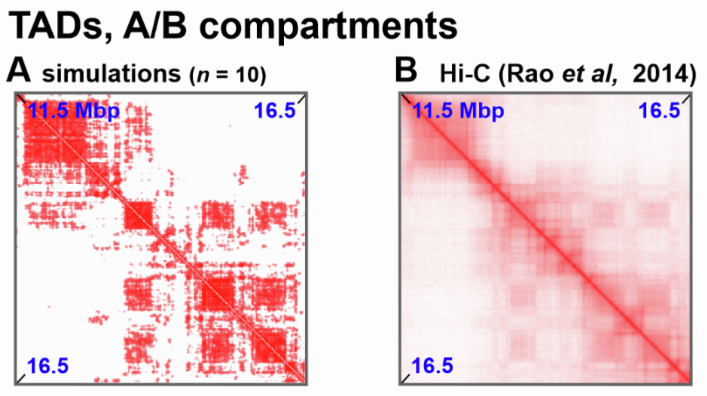

Figure S3. Simulations involving a simple polymer model yield contact maps like those found experimentally, related to Figure 1.

A. Contact map for a 5 Mbp region of HSA19 in GM12878 cells obtained from 10 polymer simulations of the whole chromosome (data from[15]).

B. Contact map of the same region obtained by Hi-C (data from[16]).

Click here to enter text.

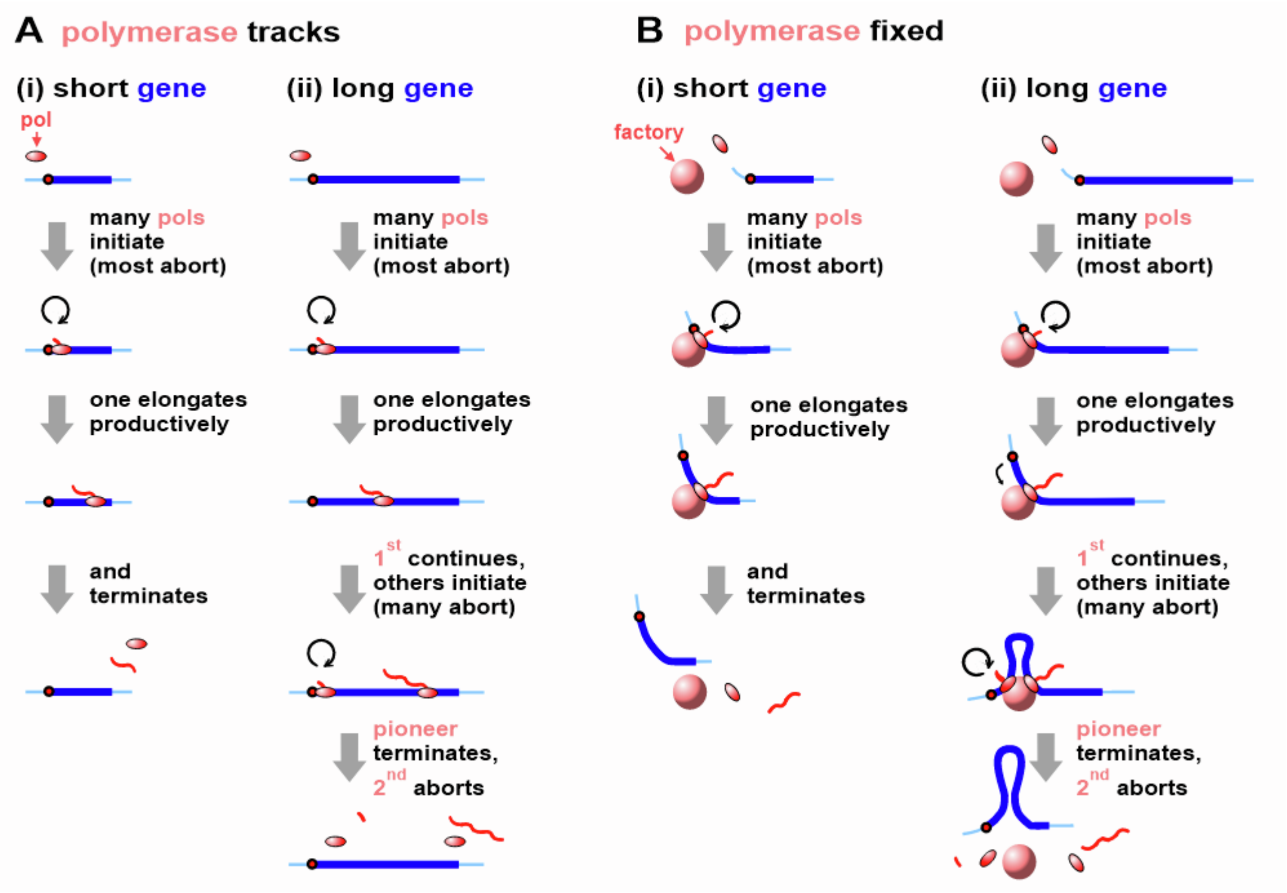

Figure S4. Transcription cycles (promoter – small red circle; polymerase – red oval; transcript – wavy red line; factory – large pink sphere), related to Figure 3.

A. Traditional model with tracking polymerases.

(**i**) Short gene. Many polymerases bind to a promoter, a few elongate for ~10 nucleotides (then many abort), and fewer still form a productive complex that tracks down the template. A variant model (not shown) has the polymerase initiating in a *hub* and tracking away from the hub[16].

(**ii**) Long gene. Now a 2nd polymerase may initiate (and probably abort) before the pioneer terminates. Few active human genes are ever loaded with >1 productively-elongating polymerase, and these so-called active genes are idle for most of their time[17].

B. Alternative with transiently-immobilized polymerases.

(**i**) Short gene. The promoter diffuses to the factory and initiates; most resulting complexes again disassemble. Once a productive complex forms, the polymerase remains transiently bound to the factory as it reels in the template and makes a transcript. As the template moves past the polymerase, close tethering restricts diffusion of the now-active template (as seen in[18]).

(**ii**) Long gene. Initial steps are as for the short gene, and yield a (productive) elongating complex that tethers the promoter close to the factory (3rd panel down); therefore, the promoter is likely to revisit the same factory (arrow) and reinitiate (as the pioneer continues to transcribe) to yield a *sub-gene loop* (4th panel down). If the 2nd polymerase aborts and the promoter cycles through several initiations and abortions, the length of this sub-gene loop grows as the pioneer continues transcribing. Then, segments lying progressively 3' are brought successively next to the promoter. 3C confirms this with various long human genes[19-21]. Super-resolution RNA FISH also confirms that nascent promoter-proximal transcripts lie next to nascent RNAs copied from progressively further into the long gene[21]. Moreover, *ChIA-Drop* shows such directional 3' bias in contacts between fly promoters and down-stream segments[22]; in this study ~80% contacts contained one genic promoter – consistent with this model applied to genomes where non-genic promoters out-number genic ones. All these results are simply explained if the active polymerase is transiently immobile, and impossible to explain if the enzyme tracks without complex additional assumptions.

Click here to enter text.

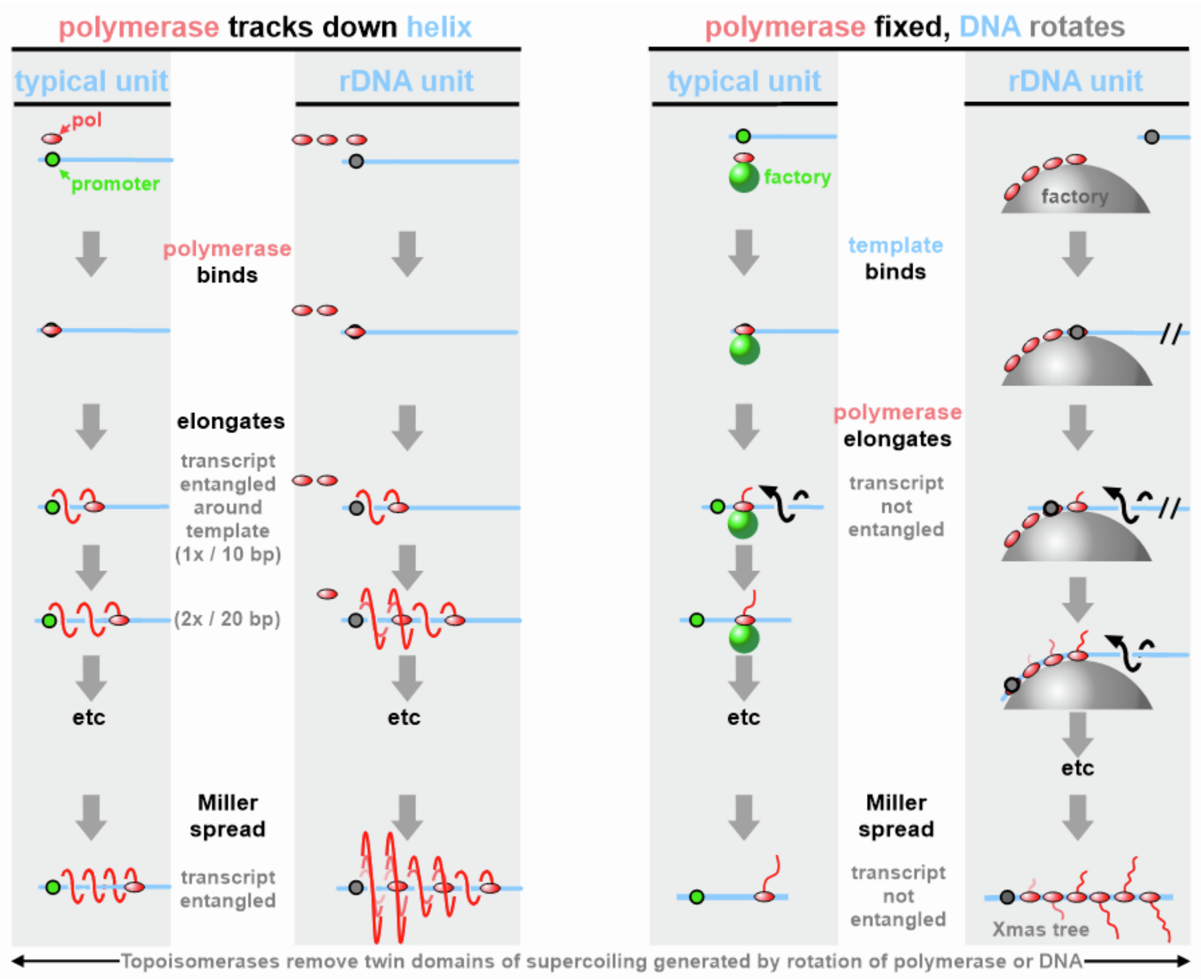

Figure S5. Interpretation of Miller spreads[23,24], related to Figure 3.
Iconic images of *Christmas trees* are often cited as the best evidence that active polymerases track down their templates. These are obtained by spreading a 3D structure with >50 polymerases tightly packed on one bacterial rDNA unit on to a 2D surface, and electron microscopy; nascent transcripts appear as *branches* in the tree (as bottom right). Most other TUs are associated with just 1 polymerase and one branch (as bottom of 3rd grey column). We argue such images[25] (and similar ones of lampbrush loops) provide strong support for our view and against the traditional one – a view confirmed by single-molecule[18,26]. Note that a distinct topological problem arises whether or not polymerases track (twin domains of supercoiling form on each side of the polymerase and are removed by topoisomerases). See also **Video 1**.
1st grey column: one polymerase tracking down a typical unit. Transcription requires lateral and rotational movement along and around a helix, so tracking along a helical path generates a transcript entwined once around a template for every 10 bp transcribed. Spreading an engaged polymerase and 500-nucleotide transcript should yield a transcript with 50 entanglements (only 3 shown at bottom) – and not one extended (un-entangled) *branch* as in Miller's images. No mechanism is known that can untwine a transcript exactly the right number of times to free it from the template to give such images.
2nd grey column: many polymerases (only 3 shown) tracking down a rDNA unit. As for a single polymerase, each transcript should become entwined many times. With >50 polymerases we should see a dense mass of RNA around the template (not distinct and untwined *branches* extending from the *trunk*).
3rd grey column: a single enzyme fixed to the green factory reeling in a typical unit. Now DNA moves laterally plus rotationally (black arrow). Screwing a bolt through a fixed nut provides an analogy. On spreading, the template is stripped off the factory, so we see a single untwined transcript.
4th grey column: many fixed enzymes (up to 6 shown) reeling in a rDNA unit. As before, the template moves laterally and rotationally (black arrows) over a larger factory to yield untwined transcripts, and now spreading yields the *Xmas trees* seen in the iconic images of single rDNA units. Here, screwing a bolt through 6 fixed nuts provides the analogy

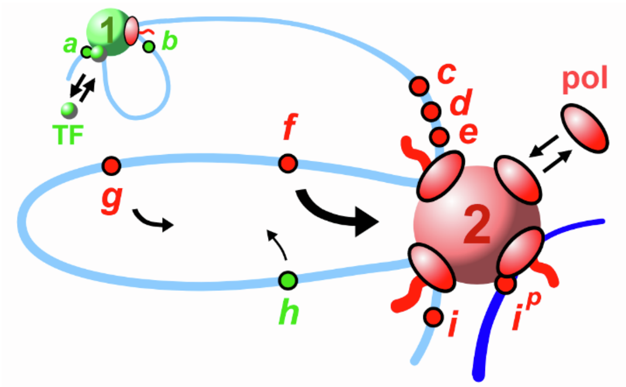

Figure S6. Regulatory motifs, related to Figure 3.
Letters mark green and red promoters that tend to initiate in numbered factories of similar color. All motifs discussed are transcribed when active.
(**i**) *Enhancer*[27-29]. Transcription of *e* ensures *f* is tethered close to red 2, so *f* often visits 2 and initiates (creating a new *e*:*f* loop and 3C contact). Then, we call *e* an enhancer of *f*.
(**ii**) *SEs*[30]. *e* detaches from 2 when its polymerase terminates. If *c*, *d*, and *e* are strong promoters, there is a good chance one re-initiates in 2 to re-tether *f* close to 2 and increase its likelihood of re-initiation. Consequently, we call *c*, *d*, and *e* a SE of *f*. Repeated initiation of *f* generates the bursty transcription seen *in vivo* and in polymer simulations[14].
(**iii**) *Silencers*[31].Transcription of *i* ties green *h* close to red 2, and far from green 1. While *h* and *f* visit red 2 equally often, *f* does not initiate there as green factors are missing; moreover, while *i* is transcribed, *h* cannot visit green 1. Consequently, we call *i* a silencer of *h*.
(iv) *Boundaries*, *insulator*[32,33]. If *b* and *c*-*e* are transcribed often, *h* visits green 1 rarely; consequently, *b* + *c*-*e* prevent *f* and *h* from diffusing further afield, and so we call them boundaries and insulators.
(**v**) *eQTLs* and *QTLs*. If a SNP in *e* reduces TF binding, this shortens the time *e* is bound to red 2 – to decrease close tethering (and so firing frequency) of *f* and *g*. If *f* and *g* are genes, we call this SNP an eQTL as it down-regulates both. As *f* and *g* bind red TFs, they are functionally related; this explains why eQTLs target (and often contact) functionally-related genes. Now consider a SNP in *i* that reduces binding to red 2. This shortens the time *i* is bound to 2 and allows *h* to visit green 1 and so fire more often. If *h* is a gene, we will call this SNP an eQTL as it up-regulates *h*. If *f*, *g*, and *h* encode proteins affecting a trait of interest, we call these SNPs in *i* and *h* QTLs. Critically both eQTLs and QTLs act co-transcriptionally here (of course, this does not exclude them from acting post-transcriptionally as in the omnigenic and other models).
(**vi**) Motifs leading to loose chromosome pairing during meiosis and transvection[34]. Homologs have similar DNA sequences and so organize similar strings of colored factories (which we will call *homologous factories*) down their lengths. If i^p is the paternal homolog of maternal *i*, homologs are tied together through red factory 2. Binding of more promoters to appropriate homologous factories will then zip chromosomes together. We suggest this occurs rarely in somatic cells, but often in meiotic ones – where complete zipping is aided by long times, plus a variety of species-specific mechanisms[35]. In human meiosis, we anticipate such a (1st) homology search depends on promoters sifting through a few thousand factories/cell to find appropriately-colored ones. Subsequently, the (2nd) well-known base-pairing search screens through thousands of bases/loop (not the billions in the genome) to achieve tight recombinational pairing (attainable in the time available, even when loops contain repeated sequences that defeat a genome-wide search). Now consider transvection – the *trans* complementation originally seen in flies where mutations on different homologs recreate the wild-type, but only if homologs pair[36]. Imagine the maternal homolog encodes wild-type gene f^m plus a mutant enhancer e^m (so f^m is silent), and the paternal one a mutant f^p plus a wild-type e^p (so f^p is also silent). Once homologs pair, wild-type f^m becomes tethered close to a red homologous factory, so enabling it to fire. This factory-based search is aided by local concentrations (in relevant fly cells homologs are polytenized >100x, and in meiosis each pairing partner is duplicated). [It is also driven by the depletion- and bridging-induced attractions that operate throughout evolutionary time, and once bi-/multi-valent proteins appear, respectively. Therefore, both could occur before the Darwinian threshold marking the transition from primordial cells (each probably with many genomes/cell), through LUCA (the last universal common ancestor), to distinct archaeal, bacterial, and eukaryotic lineages[37]. This has obvious implications for which paths evolution followed, and when/how the mystery that is sexual dimorphism developed.]

Click here to enter text.

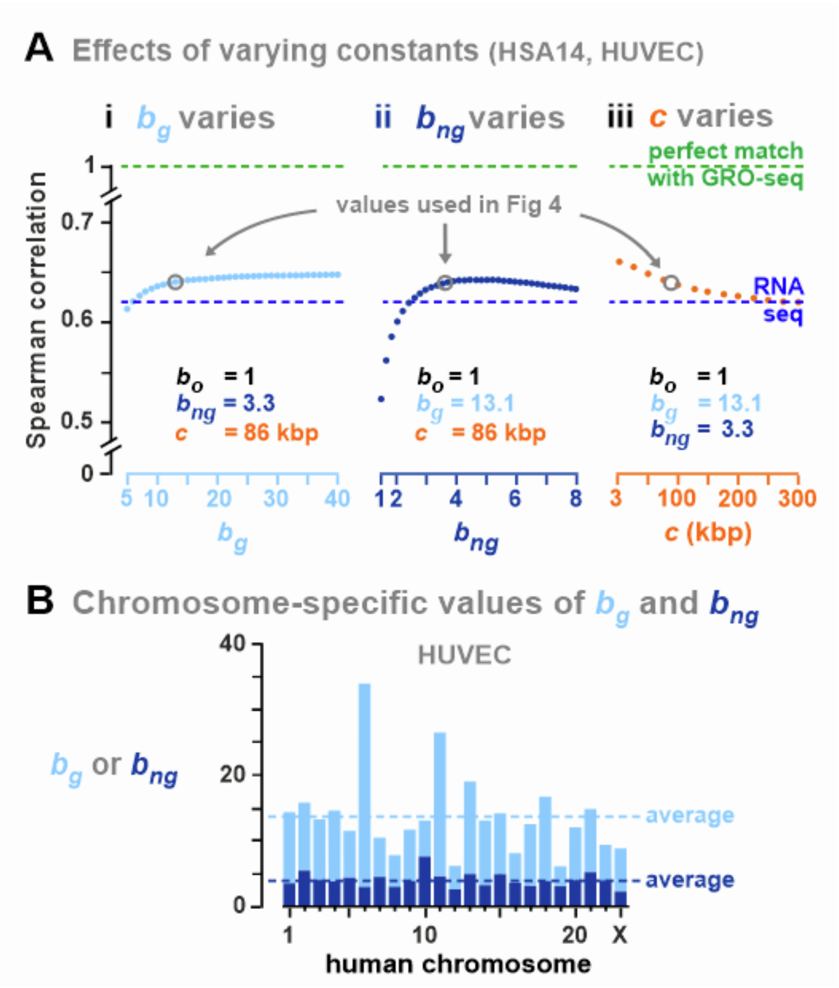

Figure S7. Artificially varying constants within experimentally-observed limits has little effect on the performance of the 3-state formula, related to Figure 4.

A. Effects of artificially varying of b_g, b_{ng}, and c (HUVEC, HSA14). The formula is applied as the value of one constant is systematically varied (invariant values indicated); then, rank orders of all promoter activities are determined, and Spearman correlations determined using GRO-seq data as a reference. Grey circles indicate values used in **Figure 4**. Spearman correlations prove to be relatively insensitive to variations of b_g and b_{ng} (within experimentally-determined limits seen in panel B), and over a wide range of possible average loop lengths.

B. Chromosomes-specific values of b_g and b_{ng} determined from GRO-seq data (HUVEC). For each chromosome, promoters are classified as b_g, b_{ng}, or b_o, the number of reads seen in GRO-seq data extracted, values normalized relative to those seen with all b_o promoters (where the average value is set equal to 1), and averages determined. Chromosome-specific values of b_g are always greater than those of b_{ng} (dotted lines give average values). For all chromosomes in the 3 cell types examined (i.e., HUVEC, GM12878, H1-hESC), values of b_g vary between 3-35, and of b_{ng} between 1-8 (not shown).

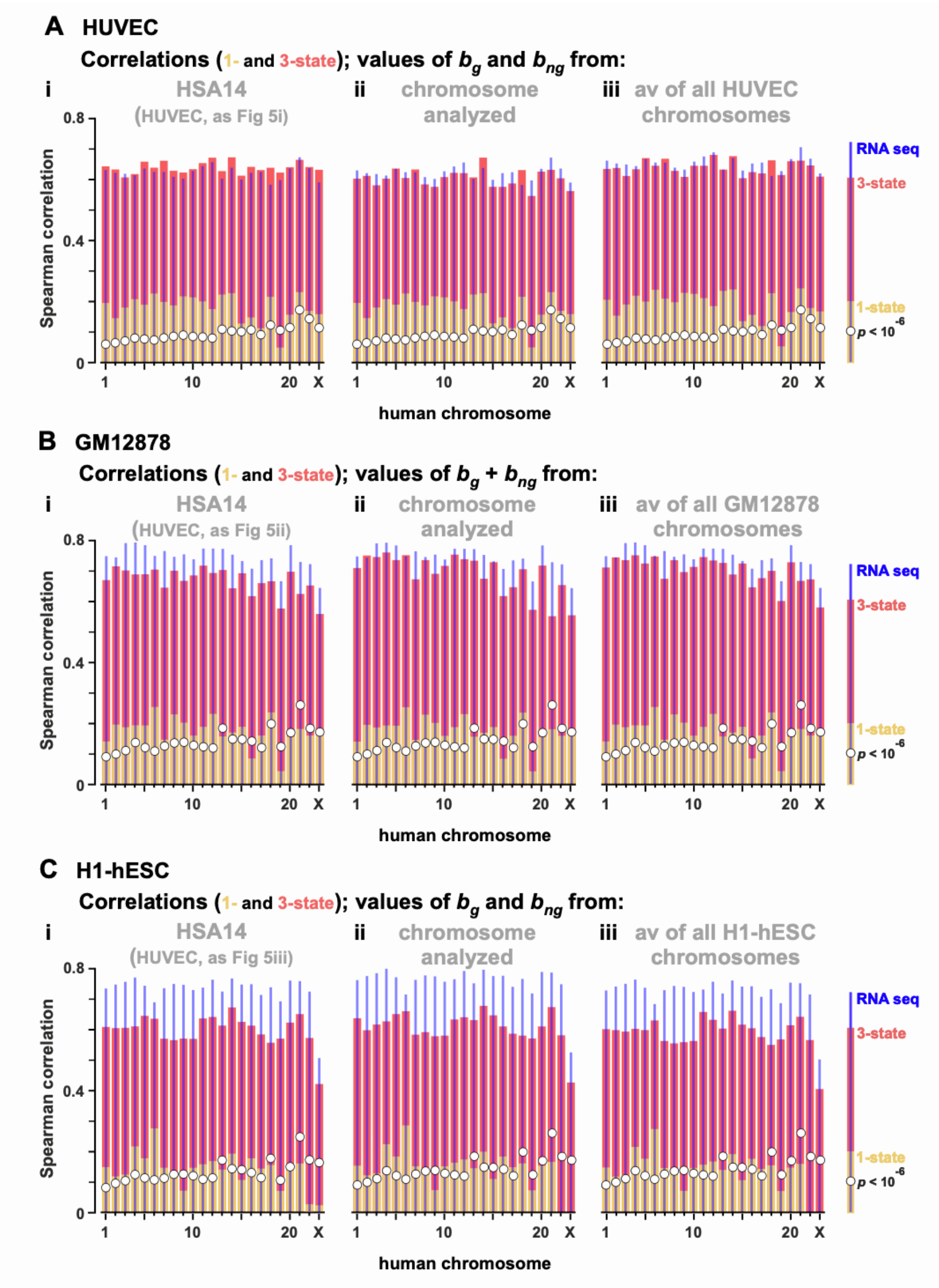

Figure S8. Varying constants b_g and b_{ng} has little effect on Spearman correlations obtained in different cell types, related to Figure 5. Predictions obtained with the 1- and 3-state formulae are compared with those obtained experimentally by RNA-seq and GRO-seq for all chromosomes in the cell. With the formulae, active promoters are identified using DHSs, their $ptrans$ values calculated and ranked from high to low, and Spearman correlations between the rank order and that from GRO-seq determined (using c=86c=86 kbp). For the 3-state formula, values of b_g and b_{ng} are derived from GRO-seq data using the chromosomes indicated. Note that the p values computed measure the likeliness that correlations are obtained by chance, hence these are identical in all panels in one row. In the 1st panel in each row, values of b_g and b_{ng} are based on those found in HSA14 (b_g = 13.1, b_{ng} = 3.3, and b_o = 1) and are reproduced from **Figure 5** to allow comparison. In the 2nd panel, they are for each individual chromosome in that cell, and applied to each chromosome individually. In the 3rd panel, the average value for all chromosomes in that cell are applied to each chromosome individually. Spearman correlations prove to be relatively insensitive to the variations tested. **A.** Results for HUVEC cells. **B.** Results for GM12878 cells. **C.** Results for H1-hESC cells.

Click here to enter text.

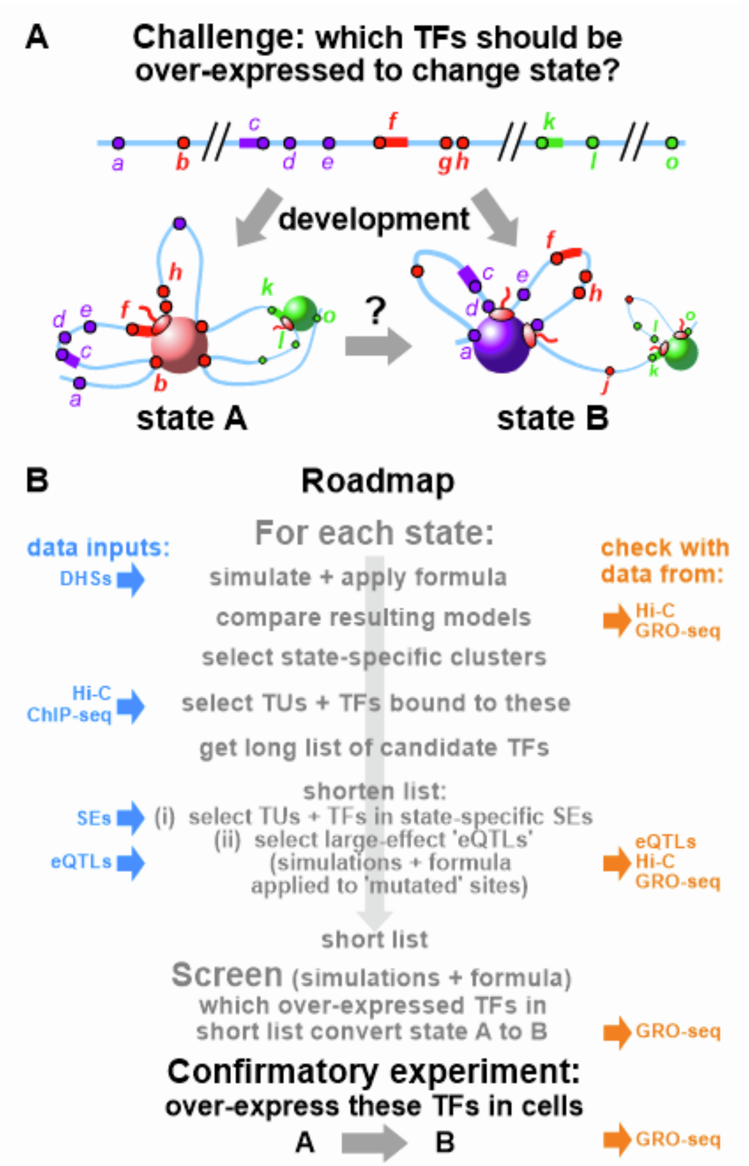

Figure S9. Solving a grand challenge: the *ab initio* determination of which TFs to over-express to switch any human cell from state A to B, related to Figure 6.
We know TFs specify cell identity by binding to cognate sites associated with unique SEs[9,38], that frequent promoter:promoter contacts form sub-maximal cliques[39,40,51], and that chromosomes in different states have their own sets of characteristic structure[41].
A. Cartoon: hypothetical genetic map and example structures found in states A + B. TUs *a-o* are in alphabetical order (genic ones – *c*, *f*, *k*; non-genic – all others). Red promoters are active in state A, purple ones in state B, and green (housekeeping) ones in both states. In this conformation of state A, red gene *f* is transcribed (*g* and *h* are its SE – both currently untranscribed), as is housekeeping-gene *k*. In state B, purple gene *c* is tethered close to the purple factory by *d* (which forms a SE with *e*) and will soon fire, and housekeeping *k* is again being transcribed. The grand challenge is to find which TFs to over-express to convert state A to B.
B. One possible roadmap. For each state, we identify all active promoters[42] (e.g., using DHS or ATAC-seq data), generate sets of 3D structures using polymer simulations (e.g., with HiP-HoP[43]) + the formula, check results (e.g., using GRO-seq, NET-seq, Hi-C, micro-C, GAM, pore-C), and select clusters uniquely present in each state. We now identify clusters in the state-specific sub-set rich in particular TFs that specialize in transcribing state-related TUs. Thus, red TUs associated with pink factories in state A will tend to contact other red TUs and so constitute a small-world network detectable by mapping close DNA:DNA contacts (e.g., using Hi-C). For example, *b*, *f*, *g*, and *h* all form frequent pairwise contacts with each other, and with other red TUs on the same and different chromosomes. This small-world of TUs should all bind the same red TFs (detected using ChIP-seq, with contacts between members of the clique being confirmed by Hi-C). This combination will probably yield one long list of red state-specific TUs/TFs, plus another for purple state-specific ones. We now shorten such lists by selecting (i) critical TFs specifying state B that we assume bind to B-specific SEs and promote transcription of B-specific/purple genes, and (ii) large-effect eQTLs affecting B-specific genes (using polymer simulations + the formula, and checking that appropriate contacts are made in each distinct small-world network) by eliminating binding to sites in each SEs in turn to see what consequential effects there are on computed transcriptional activity. Having shortened the list, we screen (using polymer simulations + the formula) to see whether *over-expressing* remaining TFs converts state A to B, and select ones inducing the wanted change. Finally, we confirm experimentally this fate switch. Obviously, the roadmap will be augmented by other approaches[41,44-50].

Click here to enter text.

SUPPLEMENTARY REFERENCES

1. Yatskevich, S., Rhodes, J., and Nasmyth, K. (2019). Organization of chromosomal DNA by SMC complexes. Annu. Rev. Genet. *53*, 445-482.
2. Davidson, I.F., and Peters, J.M. (2021). Genome folding through loop extrusion by SMC complexes. Nat. Rev. Mol. Cell Biol. *22*, 445-464
3. Ryu, J.-K., Bouchoux, C., Liu, H.W., Kim, E., Minamino, M., de Groot, R., Katan, A.J., Bonato, A., Marenduzzo, D., Michieletto, D., *et al*. (2021). Bridging-induced phase separation induced by cohesin SMC protein complexes. Science Advances *7*, eabe5905.
4. Gu, B., Comerci, C.J., McCarthy, D.G., Saurabh, S., Moerner, W.E., and Wysocka, J. (2020). Opposing effects of cohesin and transcription on CTCF organization revealed by super-resolution imaging. Mol. Cell *80*, 699-711.
5. Boehning, M., Dugast-Darzacq, C., Rankovic, M., Hansen, A.S., Yu, T., Marie-Nelly, H., McSwiggen, D.T., Kokic, G., Dailey, G.M., Cramer, P., *et al.* (2018). RNA polymerase II clustering through carboxy-terminal domain phase separation. Nature Struct. Mol. Biol. *25*, 833-840.
6. Lu, H., Yu, D., Hansen, A.S., Ganguly, S., Liu, R., Heckert, A., Darzacq, X., and Zhou, Q. (2018). Phase-separation mechanism for C-terminal hyperphosphorylation of RNA polymerase II. Nature *558*, 318-323.
7. Plys, A.J., and Kingston, R.E. (2018). Dynamic condensates activate transcription. Science *361*, 329-330.
8. Shao, W., Bi, X., Pan, Y., Gao, B., Wu, J., Yin, Y., Liu, Z., Zhang, W., Jiang, X., Ren, W., *et al.* (2021). Phase separation of RNA-binding protein promotes polymerase engagement and transcription. Nat. Chem. Biol. *18*, 70-80.
9. Boija, A., Klein, I.A., Sabari, B.R., Dall'Agnese, A., Coffey, E.L., Zamudio, A.V., Li, C.H., Shrinivas, K., Manteiga, J.C., Hannett, N.M., *et al*. (2018). Transcription factors activate genes through the phase-separation capacity of their activation domains (2018). Cell *175*, 1842-1855.
10. Ferrie, J.J., Karr, J.P., Tjian, R., and Darzacq, X. (2022). *Structure*-function relationships in eukaryotic transcription factors: The role of intrinsically disordered regions in gene regulation. Mol. Cell *82*, 3970-3984.
11. Shin, Y., Chang, Y.-C., Lee, D.S.W., Berry, J., Sanders, D.W., Ronceray, P., Wingreen, N.S., Haataja, M., and Brangwynne, C.P. (2018). Liquid nuclear condensates mechanically sense and restructure the genome. Cell *175*, 1481-1491.
12. Marenduzzo, D., Finan, K., and Cook, P. R. (2006). The depletion attraction: an underappreciated force driving cellular organization. J. Cell Biol. *175*, 681-686.
13. Mitchison, T.J. (2019). Colloid osmotic parameterization and measurement of subcellular crowding. Mol. Biol. Cell *30*, 173-180.
14. Brackley, C. A., Johnson, J., Kelly, S., Cook, P. R., and Marenduzzo, D. (2016). Simulated binding of transcription factors to active and inactive regions folds human chromosomes into loops, rosettes and topological domains. Nucleic Acids Res. *44*, 3503-3512.
15. Rao, S.S.P., Huntley, M.H., Durand, N.C., Stamenova, E.K., Bochkov, I.D., Robinson, J.T., Sanborn, A.L., Machol, I., Omer, A.D., Lander, E.S., *et al*. (2014). A 3D map of the human genome at kilobase resolution reveals principles of chromatin looping. Cell *159*, 1665-1680.
16. Cramer, P. (2019). Organization and regulation of gene transcription. Nature *573*, 45-54.
17. Larsson, A.J.M., Johnsson, P., Hagemann-Jensen, M., Hartmanis, L., Faridani, O.R., Reinius, B., Segerstolpe, A., Rivera, C.M., Ren, B., and Sandberg, R. (2019). Genomic encoding of transcriptional burst kinetics. Nature *565*, 251-254.
18. Nagashima, R., Hibino, K., Ashwin, S.S., Babokhov, M., Imai, S.F.R., Nozaki, T., Tamura, S., Tani, T., Kimura, H., Shribak, M., Kanemaki, M.T., Sasai, M., and Maeshima, K. (2019). Single nucleosome imaging reveals loose genome chromatin networks via active RNA polymerase II. J. Cell Biol. *218*, 1511-1530.
19. Papantonis, A., Larkin, J.D., Wada, Y., Ohta, Y., Ihara, S., Kodama, T., and Cook, P.R. (2010). Active RNA polymerases: mobile or immobile molecular machines? PLoS Biol. *8*, e1000419.
20. Larkin, J.D., Cook, P.R., and Papantonis, A. (2012). Dynamic reconfiguration of long human genes during one transcription cycle. Mol. Cell. Biol. *32*, 2738-2747.
21. Larkin, J.D., Papantonis, A., Cook, P.R., and Marenduzzo, D. (2013). Space exploration by the promoter of a long human gene during one transcription cycle. Nucl. Acids Res. *41*, 2216-2227.
22. Zheng, M., Tian, S.T., Capurso, D., Kim, M., Maurya, R., Lee, B., Piecuch, E., Gong, L., Zhu, J.J., Li, Z., *et al*. (2019). Multiplex chromatin interactions with single-molecule precision. Nature *566*, 558-562.
23. Miller, O.L., and Bakken, A.H. (1972). Morphological studies of transcription. Acta Endocrinol. Suppl. (Copenh). *168*, 155-177
24. Miller, O.L., Hamkalo, B.A., and Thomas, C.A. (1970). Visualization of bacterial genes in action. Science *169*, 392-395.
25. Papantonis, A., and Cook, P.R. (2013). Transcription factories; genome organization and gene regulation. Chem. Rev. *113*, 8683-8705.
26. Ide, S., Imai, R., Ochi, H., and Maeshima, K. (2020). Transcriptional suppression of ribosomal DNA with phase separation. Science Advances *6*, eabb5953.

Click here to enter text.

27. Andersson, R., and Sandelin, A. (2020). Determinants of enhancer and promoter activities of regulatory elements. Nature Revs Genetics *21*, 71-87.
28. Schoenfelder, S., and Fraser, P. (2019). Long-range enhancer-promoter contacts in gene expression control. Nat. Rev. Genet. *20*, 437-455.
29. Furlong, E.E.M., and Levine, M. (2018). Developmental enhancers and chromosome topology. Science *361*, 1341-1345.
30. Hnisz, D., Shrinivas, K., Young, R. A., Chakraborty, A. K., and Sharp, P. A. (2017). A phase separation model for transcriptional control. Cell *169*, 13-23.
31. Pang, B., van Weerd, J.H., Hamoen, F.L., and Snyder, M.P. (2023). Identification of non-coding silencer elements and their regulation of gene expression. Nature Revs Mol. Cell Biol. *24*, 383-395.
32. Hsieh T.S., Cattoglio, C., Slobodyanyuk, E., Hansen, A.S., Rando, O.J., Tjian, R., and Darzacq, X. (2020). Resolving the 3D landscape of transcription-linked mammalian chromatin folding. Mol Cell. *78*, 539-553.
33. Krietenstein, N., Abraham, S., Venev, S.V., Abdennur, N., Gibcus, J., Hsieh, T.-H.S., Parsi, K.M., Yang, L., Maehr, R., Mirny, L.A., *et al.* (2020). Ultrastructural details of mammalian chromosome architecture. Mol. Cell *78*, 554-565.E7.
34. Xu, M., and Cook, P.R. (2008). The role of specialized transcription factories in chromosome pairing. Biochim. Biophys. Acta *1783*, 2155-2160.
35. Kim, H.J., Liu, C., and Dernburg, A.F. (2022). How and why chromosomes interact with the cytoskeleton during meiosis. Genes *13*, 901.
36. Fukaya, T., and Levine, M. (2017). Transvection. Curr. Biol. *27*, R1047-R1049.
37. Woese, C.R. (2002). On the evolution of cells. Proc. Natl. Acad. Sci. USA *99*, 8742-8747.
38. Whyte, W.A., Orlando, D.A., Hnisz, D., Abraham, B.J., Lin, C.Y., Kagey, M.H., Rahl, P.B., Lee, T.I., and Young, R.A. (2013). Master transcription factors and mediator establish super-enhancers at key cell identity genes. Cell *153*, 307-319.
39. Choy, M.K., Javierre, B.M., Williams, S.G., Baross, S.L., Liu, Y., Wingett, S.W., Akbarov, A., Wallace, C., Freire-Pritchett, P., Rugg-Gunn, P.J., *et al.* (2018). Promoter interactome of human embryonic stem cell-derived cardiomyocytes connects GWAS regions to cardiac gene networks. Nat. Commun. *9*, 2526.
40. Liu, S., Chen, H., Ronquist, S., Seaman, L., Ceglia, N., Chen, P.Y., Higgins, G., Baldi, P., Smale, S., Hero, A., *et al*. (2018). Genome architecture mediates transcriptional control of human myogenic reprogramming. iScience *6*, 232-246.
41. Di Stefano, M., Stadhouders, R., Farabella, I., Castillo, D., Serra, F., Graf, T., and Marti-Renom, M.A. (2020). Transcriptional activation during cell reprogramming correlates with the formation of 3D open chromatin hubs. Nat. Commun. *11*, 2564.
42. Zhang, K., Hocker, J.D., Miller, M., Hou, X., Chiou, J., Poirion, O.B., Qiu, Y., Li, Y.E., Gaulton, K.J., Wang, A., Preissl, S., and Ren, B. (2021). A single-cell atlas of chromatin accessibility in the human genome. Cell *184*, 5985-6001.
43. Buckle, A., Brackley, C.A., Boyle, S., Marenduzzo, D., and Gilbert, N. (2018). Polymer simulations of heteromorphic chromatin predict the 3D folding of complex genomic loci. Mol. Cell *72*, 786-797.
44. Avsec, Z., Agarwal, V., Visentin, D., Ledsam, J.R., Grabska-Barwinska, A., Taylor, K.R., Assael, Y., Jumper, J., Kohli. P., and Kelley, D.R. (2021). Effective gene expression prediction from sequence by integrating long-range interactions. Nature Methods *18*, 1196-1203.
45. Ronquist, S., Patterson, G., Muir, L.A., Lindsly, S., Chen, H., Brown, M., Wicha, M.S., Bloch, A., Brockett, R., and Rajapakse, I. (2017). Algorithm for cellular reprogramming. Proc. Natl. Acad. Sci. *114*, 11832-11837.
46. Cahan, P., Li, H., Morris, S.A., da Rocha, E.L., Daley, G.Q., and Collins, J.J. (2014). CellNet: network biology applied to stem cell engineering. (2014). Cell *158*, 903-915.
47. Rackham, O.J.L., Firas, J., Fang, H., Oates M.E., Holmes, M.L., Knaupp, A.S., The FANTOM consortium, Suzuki, H., Nefzger, C.M., Daub, C.O., *et al*. (2016). A predictive computational framework for direct reprogramming between human cell types. Nat. Genet. *48*, 331-335.
48. Dunn, S.J., Li, M.A., Carbognin, E., Smith, A., and Martello, G. (2019). A common molecular logic determines embryonic stem cell self-renewal and reprogramming. EMBO J. *38*, e100003.
49. Liu, X., Ouyang, J.F., Rossello, F.J., Tan, J.P., Davidson, K.C., Valdes, D.S., Schröder, J., Sun, Y.B.Y., Chen, J., Knaupp, A.S., *et al*. (2020). Reprogramming roadmap reveals route to human induced trophoblast stem cells. Nature *586*, 101-107.
50. Kamimoto, K., Stringa, B., Hoffmann, C.M., Jindal, K., Solnica-Krezel. L., and Morri, S.A. (2023). Dissecting cell identity via network inference and in silico gene perturbation. Nature *614*, 742-751.
51. Dotson, G.A., Chen, C., Lindsly, S., Cicalo, A., Dilworth, S., Ryan, C., Jeyarajan, S., Meixner, W., Stansbury, C., Pickard, J., *et al*. (2022). Deciphering multi-way interactions in the human genome. Nature Comm. *13*, 5498.
